# Evaluating access to psychosocial services for the medicaid-insured children in Georgia

**DOI:** 10.1186/s12889-025-21374-7

**Published:** 2025-01-20

**Authors:** Yujia Xie, Pravara Harati, Janani Rajbhandari-Thapa, Nicoleta Serban

**Affiliations:** 1 Georgia Institute of Technology, H. Milton Stewart School of Industrial and Systems Engineering, 755 Ferst Dr NW, Atlanta, 30332 Georgia; 2 Georgia Department of Public Health, Atlanta, Georgia; 3 Department of Health Policy and Management, University of Georgia, Athens, 30602 Georgia

**Keywords:** Healthcare access, Psychosocial services, Pediatric healthcare, Medicaid

## Abstract

**Background:**

Evaluating access to psychosocial services can inform policy decision-making on ways to address shortages in the availability of mental health (MH)-specialized providers. The objective of the study was to assess how the mental health (MH)-specialized workforce met the demand for psychosocial services of Medicaid-insured children in Georgia, with direct relevance in establishing quantitative network adequacy.

**Methods:**

We used the 2018 Medicaid (TAF) claims data, the 2018 National Plan and Provider Enumeration System database, and the 2019 Georgia school-based program data to estimate community-level demand and practice-level supply of psychosocial services. We evaluated the availability of services using mathematical models. The outcome measures were met demand and travel distance. We explored the impact of increasing in-home care delivery, expanding Medicaid participation, or increasing caseload for the Medicaid-participating providers on improving met demand for psychosocial services.

**Results:**

Our findings showed that 34% of the demand from Medicaid-insured children in Georgia remained unmet, and 25% of the Georgia census tracts (rural 79%; urban 16%) had < 50% service coverage. The travel distance for in-clinic services was 3.84 miles on average. Increasing provider Medicaid caseload or expanding Medicaid participation, resulting in a 5–40% supply increase, would reduce unmet demand to 7% and decrease the number of unserved and underserved census tracts to 3% and 2% respectively. Meeting 75% of the demand required a 15% increase in the supply.

**Conclusions:**

The main source of network inadequacy was the scarcity of MH providers available to Medicaid-insured children in Georgia, coming from both the limited caseload of existing MH providers and low Medicaid participation, rather than travel constraints. Increasing provider caseload and expanding Medicaid participation were found to reduce unmet demand. Interventions increasing caseloads were the most effective intervention since existing Medicaid-participating providers already had sufficient network coverage geographically.

**Supplementary Information:**

The online version contains supplementary material available at 10.1186/s12889-025-21374-7.

## Introduction

The demand for mental health (MH) services among low-income children, specifically those insured by Medicaid, has increased while supply shortage has persisted [1]. The United States Health Resources and Services Administration projected a shortage of at least 50,000 MH providers by 2030 nationwide [2]. The workforce shortage has been cited as a primary access barrier to psychosocial services [3–5]. Given its role of providing insurance coverage for tens of millions of children, Medicaid has become a substantive source of MH services for children. However, Medicaid faces limited availability of providers participating in the program, translating into long wait times for patients to receive service [6]. Provider shortage has been further compounded by transportation challenges and lack of parents’ availability to take time-off work, often resulting in inadequate MH treatment or forgoing MH care altogether.

To overcome challenges in healthcare access, particularly for psychosocial services, federal and state policies require health systems to establish network adequacy standards to ensure service providers are accessible (e.g., reasonable travel distance) and available (e.g., reasonable wait time) MH providers [6, 7]. A recent change in the federal rule replaced requirements of access standards, with guidelines on *quantitative network adequacy (QNA)* [8]. The QNA rule required Medicaid managed care plans to ensure that they have capacity to serve in their service area and maintain a sufficient geographic distribution of providers [6]. Because QNA is a state-level requirement for maintaining an adequate network, under this policy change, states need to develop state-specific approaches for QNA; however, there was insufficient guidance on what might be deemed ‘adequate’.

National or state-based studies that serve as a model to evaluate QNA were lacking in the MH literature. In this study, we aimed to investigate how and to what extent Medicaid-insured children diagnosed with prevalent pediatric MH conditions obtained necessary psychosocial services from existing MH-specialized workforce (i.e., have healthcare access). Studies such as this are needed to subsequently identify policy interventions to improve systemic barriers to accessing MH care [9, 10]. We thus focus on the *availability* of MH-specialized workforce to provide care to Medicaid-insured children as the primary access measure of interest [9, 11], with direct relevance to establishing QNA.

We applied mathematical models to estimate the (un)met demand of Medicaid-insured children for psychosocial services delivered by the existing MH-specialized workforce available to Medicaid programs. This study focused on access to psychosocial services for the state of Georgia, which is a large, racially diverse state with more than 1.2 million Medicaid-enrolled children [12, 13]. Georgia Medicaid along with the Department of Behavioral Health and Developmental Disabilities (DBHDD) have provided MH benefits to many children in need of care, with recent investments in community-based MH care in response to the change in the federal policy amending the free care rule in 2015 and expanded in 2017 [14, 15]. Given such policies, we also evaluated hypothetical but potential workforce interventions to improve supply of service providers for improving access to MH care using statistical approaches. While this study focuses on only one state, the approaches provided can be implemented for other states.

## Methods

### Data sources

Data sources included the 2018 Medicaid claims data acquired from the Centers of Medicare and Medicaid Services (CMS), the 2018 National Plan and Provider Enumeration System (NPPES) database, and the 2019 DBHDD-funded, Georgia School-based Program (APEX program) data on school-based psychosocial services throughout Georgia [16].

This study was approved by the Georgia Tech Institutional Review Board. Additional details are in Figure A1 in Online-Supplement A, including descriptions of data imputation addressing the cell size restrictions in the data use agreement to mask results with cell size less than or equal to 10 observations (referred to as the 11-cell rule).

### Study population

The study population consisted of Medicaid-enrolled children (aged 3–18) with at least one Medicaid claim for psychosocial services or at least two claims with MH diagnoses recorded on different dates in 2018 in Georgia. Children under age three were excluded because of challenges in establishing MH diagnosis at that age. We did not exclude children from the study population based on enrollment duration, but we weighted the sampling for deriving census-tract demand based on 2018 Medicaid enrollment, thus considering prevalence adjusted for enrollment length. *MH diagnoses* were identified based on ICD-10 codes for MH conditions (See Online-Table A1.) *Psychosocial services* were identified using CPT and HCPCS codes for assessment, psychotherapy, psychoeducation, and psychosocial rehabilitation. To ensure all relevant codes were represented, codes were determined by identifying all codes accompanying child MH diagnoses claims, selecting the 95% most frequently appearing, and removing any found irrelevant after consultations with practicing clinicians (See Online-Table A2.)

### Census-tract demand derivation

Demand for psychosocial services was measured in the unit of *visits*, each of which was the aggregation of all claims belonging to the *same patient* submitted by the *same provider* within the *same date*. Further, we defined *realized demand* as visits *received* by (delivered to) MH-diagnosed children, and *potential demand* as visits *requested although not all necessarily received* by MH-diagnosed children. We assumed that all MH-diagnosed children demand/request psychosocial services, even if for assessment only. However, Medicaid claims only captured realized demand, whereas *potential demand* was more appropriate for evaluating healthcare access because it captured both the realized visits as well as psychosocial services that were requested but not received following a MH diagnosis. We considered the demand instead of the *need* (i.e., appropriate care specified by recommended care guidelines) of MH care because the lack of specific guidelines for all prevalent pediatric-MH conditions made the estimation of needs challenging [17].

We derived the potential demand for psychosocial services within each census tract for three age groups (pre-school: 3–4; children: 5–12; adolescents: 13–18) from 2018 Medicaid data using a three-step approach:

1. Estimating the number of MH-diagnosed children (*MH prevalence*) within each census tract and age group.
2. Fitting an empirical distribution on the number of *realized* psychosocial visits per-child differentiated by age group, where the distribution assumed at least two visits per-child per-year.
3. Sampling the *potential* demand (hereafter referred to as *demand*) for every MH-diagnosed child in each census tract from the age-appropriate fitted distribution derived in Step 2.

The zip code is the smallest geographic unit of enrollees’ residence available in claims data; however, not an appropriate proxy for community-level outcomes. Step 1 employed a randomized geo-imputation method [18], projecting MH prevalence from the zip code to the census tract level, using a population-based weighting method and using the crosswalk between zip codes and census tracts.

Step 2 considered data from children with at least two realized visits to account for both assessment and treatment visits. The fitted distributions were modified to be capped at their 95th percentiles, representing more than 49, 60, 64 visits/year for pre-school, children, and adolescents age groups respectively. Children requiring a higher frequency of care (those above the 95th threshold) are likely to need specific specialized care outside the settings studied in this study. Online Figure A2 provides the flow chart of census-tract demand derivation.

Census tracts were further classified as urban or rural (*urbanicity-rurality classification*) using Rural-Urban Commuting Area Codes (RUCAs) [19] (Online Table A3).

### Practice-level supply derivation

Practice-level *supply* represented the number of psychosocial visits (hereafter referred to as *caseloads*) available for Medicaid-insured children and from providers practicing at each *unique provider address* (hereafter referred to as *MH practice*) under three *care settings*: in-home, in-school, and in-clinic. Delivery of services under different care settings exhibited distinct characteristics, requiring estimating caseloads under each care setting separately. Similarly, to estimate demand for psychosocial services, we differentiated between realized and potential supply where *realized supply* consisted of visits that MH practices provided to MH-diagnosed children and *potential supply* consisted of visits that the practices had available.

The in-home and in-clinic realized caseloads were derived from the 2018 Medicaid claims following a multi-step procedure:

1. Obtaining the number of realized in-clinic/home caseloads delivered to children by each provider, identified by National Provider Identifier (NPI), using Medicaid claims with relevant CPT codes and with in-home or in-clinic related service codes (See Online Figure A3 and Figure A4 for details).
2. Matching the NPI with information available in the NPPES data to identify provider’s practice address. (See Online Figure A5 for details)
3. Aggregating the caseloads by case settings (in-clinic or in-home) and by unique practice address (*MH practice* herein).

The in-school *realized* caseload was obtained from the 2019 APEX data, which included the list of participating schools with corresponding realized caseloads. We treated each *school’s address* as a practice address and aggregated its caseloads across the entire year. We assumed all in-school visits in Georgia were provided through this program as it was the only statewide program delivering psychosocial services in schools during the study period [16]. See Online Figure A6 for additional details.

To derive potential caseloads, we additionally considered no-show-up and other available visits that were not delivered by adding 10% to the estimated realized caseloads. We assumed MH practices did not have additional capacity to accommodate Medicaid-insured children beyond these additional no-show-up visits.

Each MH practice was further categorized into two groups (Mental Health Entity 1 Provider or *MHE1*, Mental Health Center or *MHC*) based on its entity type and NPI specializations (See Online-Table A4).

Although Medicaid claims were used to derive realized caseload, being measured in the unit of visits delivered and submitted for reimbursement, the access modeling considered potential caseload, similar to demand, thus capturing potential access.

### Access model

In healthcare, access modeling often refers to the various methods and strategies utilized to analyze and improve how patients with demand (e.g., census-tract demand for psychosocial services) access needed health services (e.g., practice-level caseloads) [9–11]. In our case, we modeled MH access using an optimization model (*access model* herein), matching practice-level caseloads with census-tract demand for psychosocial services.

The optimization-based access model provided several advantages over traditional access methods, for example, distance to nearest service and population-to-provider ratios and gravity modeling measures [20]. The optimization-based access model captured different characteristics such as when providers delivered services in different settings (clinic, school, or home). It also incorporated different community-based barriers as children traveled for in-clinic services such as limiting the maximum travel distance from children in urban/rural census tracts. The optimization model also accounted for a wide range of scenarios or interventions by adjusting constraints, and solving such problem returned decision variables that optimized the model objective. The objective was to optimize the overall availability of the MH-specialized providers in such a way that children and their parents travel minimally to reach a MH practice available to provide care, assuming zero travel for in-home and in-school care settings. This objective was designed to measure accessibility, from which we could subsequently compare and evaluate access to psychosocial services across different settings.

The model focused on matching the available caseload of in-clinic and in-home practices to demand. In-school visits were directly assigned to each school’s nearby census tracts (within 5 miles), proportional to census tract population, to reflect the student populations attending those schools.

The access model incorporated additional system constraints to ensure the model was realistic, for example, the served caseloads to each census tract not exceeding demand, and the assigned caseloads from each practice not exceeding its capacity for each care setting. For in-clinic practices, we assumed children were located at the (population) census centroids and traveled to providers located at practice addresses. We restricted the travel distance between practice address and census tract centroids to be within 45 miles for rural census tracts and 30 miles for urban based on recommended access standards [7, 21]. We used ArcGIS to compute corresponding travel distances. In-home practices, we assumed providers delivered services to children located in a restricted list of counties that providers could serve and cover (see Online Figure A8). The constraints considered in the access model accounted for other access dimensions such as affordability (e.g. focusing on the Medicaid program), accessibility (e.g. minimizing travel distance) and accommodation (e.g. considering different care settings) [10]. See Online-Supplement B for details on the implementation of the access model.

### Outcome measures

The following outcome measures were used in the access model:

1. *Travel distance*: Average one-way travel (in miles) to receive psychosocial services as determined by the output provider and care setting assignments (0 miles for in-home and in-school visits; the distance between a patient’s census tract centroid and their provider’s practice location for in-clinic visits) (see Figure B1 for illustrations on the computation of travel distance).
2. *Percent-met demand*: The percentage of demand met by the caseloads assigned to MH practices (see Figure B2 for illustrations on the computation of percent-met demand).
3. *Service coverage*: Categorization of census tracts into (1) *Unserved* tracts with 0% met demand; (2) *Underserved* tracts with 50% or less percent-met demand (but not zero); (3) *Served tracts*, the remainder tracts (see Figure B3 for illustrations on the computation of service coverage).

Outcomes were analyzed by urbanicity-rurality and care setting.

### Intervention analysis

We considered three interventions to lower the barriers for MH-diagnosed children accessing psychosocial services.

#### Caseload intervention

This intervention aimed to increase available capacity by sampling a percentage (*ratio* herein) of existing MH practices (by 5–40%) and duplicating their caseloads. Two approaches were used to sample the subset: (1) Randomly selecting providers (*random approach*, See Online-Figures B4); and (2) Within proximity to unserved or underserved tracts (*targeted approach*, See Online-Figure B5).

#### Workforce intervention

This intervention aimed to expand the provider network by increasing MH provider participation in Medicaid. We sampled a percentage (*ratio* herein) of non-participating MH practices to accept Medicaid-insured children with varying levels of increase in participation (by 5–40%) with in each of the three care settings studied. Sampling was performed using the random and targeted approaches, illustrated in Online-Figures B6 and B7 respectively.

#### In-home intervention

This intervention targeted lowering travel distance by randomly selecting a percentage (*ratio* herein) of existing in-clinic providers (by 5–40%) and changing their service type to in-home. (See Online-Figure B8.)

We defined a *scenario* as one intervention applied for one sampling approach (random vs. targeted), in total five scenarios. The ratio described in scenario was increased from 5 to 40% to produce trends, capturing the potential access improvement with changes in the intervention percent change.

## Results

### Demand derivation

*Online-Table C1* and *Online Figures C1-C3* provides details on the study population. We identified 1,337,371 Medicaid-insured children in Georgia, with 21% of them having at least one MH diagnosis. We observed 2,095,013 psychosocial visits from 287,206 MH-diagnosed children using the 2018 Medicaid claims. On average, preschool children (aged 3–4) demanded 3.2 visits per year, lower compared to those aged 5–12 (8.2) and 13–18 (7.0).

*Online-Figure C4* displays the enrollment data; 91% of the children in the study population are enrolled for the entire year, with older children having higher rates of enrollment.

*Online-Figure C5* and *Online-Table C2* present the estimated demand. Using the modeling approach, we estimated 2,325,020 visits, 11% higher than the realized demand. 80% of the study population resided in urban census tracts, yielding 85% of the total estimated demand.

### Supply derivation

*Online-Figure C6* and *Online-Table C3* present summaries of practice-level supply. We identified 20,855 MH-specialized NPIs at 10,725 addresses throughout Georgia, with 13% accepting Medicaid, providing services at 2112 unique practice locations, and providing 85% in-clinic and 14% in-home visits. The majority (83%) of the providers were Entity-1 (individual) NPIs (*MHE1* category herein), with 51.65% of the in-clinic caseload. The entity-2 (organization) providers (*MHE2* category herein) delivered 92.01% of the caseloads served in-home care.

### Access outcome measures and inference

Table 1 provides details on the travel distance under baseline (no intervention). The travel distance for all in-clinic services was 3.84 miles on average. It was higher for children in rural (6.84) than in urban tracts (3.6).

**Table 1:** Access results (difference from baseline). Outcome measures (travel distance, percent-met demand, service coverage) from three interventions (caseload, workforce, and in-home interventions) under random and/or targeted sampling approaches with 5–40% increase. Numbers displayed as differences from baseline (no intervention)

Intervention	Sampling approach	Sampling ratio	Travel distance PER visit (miles)	Percent-met demand (%)	Unserved tract (%)	Underserved tract (%)
**No intervention (Baseline)**			**3.84**	**66.00**	**11.00**	**14.00**
Caseload	Random	5	0.29	4.00	-1.00	-1.00
		10	0.34	7.00	-2.00	-3.00
		15	0.83	10.96	-3.00	-5.00
		20	0.81	14.00	-3.00	-7.00
		25	0.84	17.00	-3.00	-9.00
		30	1.25	20.81	-4.00	-10.00
		35	1.43	23.98	-5.00	-11.00
		40	1.48	27.00	-7.00	-12.00
	Targeted	5	0.46	4.00	-1.00	-2.00
		10	0.48	7.00	-2.00	-3.00
		15	0.57	10.00	-2.00	-4.00
		20	0.98	13.86	-3.00	-6.00
		25	1.06	17.00	-4.00	-9.00
		30	1.15	20.00	-4.00	-10.00
		35	1.21	23.00	-5.00	-11.00
		40	1.68	26.75	-7.00	-12.00
Workforce	Random	5	0.31	3.52	0.00	-2.00
		10	0.10	6.52	-1.00	-3.00
		15	0.06	9.52	-1.00	-5.00
		20	0.42	13.52	-2.00	-7.00
		25	0.64	16.52	-3.00	-9.00
		30	0.67	19.52	-3.00	-10.00
		35	1.22	23.52	-5.00	-11.00
		40	1.31	26.52	-7.00	-12.00
	Targeted	5	0.20	3.52	0.00	-2.00
		10	0.15	6.52	-2.00	-3.00
		15	0.24	9.52	-2.00	-4.00
		20	0.38	13.52	-3.00	-6.00
		25	0.28	16.52	-4.00	-8.00
		30	0.39	19.52	-5.00	-9.00
		35	0.76	23.52	-6.00	-11.00
		40	0.84	26.52	-8.00	-11.00
In-Home	Random	5	-0.02	0.00	0.00	-1.00
		10	-0.05	0.00	-1.00	2.00
		15	0.18	-0.01	-1.00	0.00
		20	-0.17	0.00	0.00	3.00
		25	-0.56	-1.00	0.00	1.00
		30	-0.61	-1.00	0.00	0.00
		35	-0.84	-1.00	0.00	1.00
		40	-0.57	-1.00	0.00	0.00

**Fig. 1:**
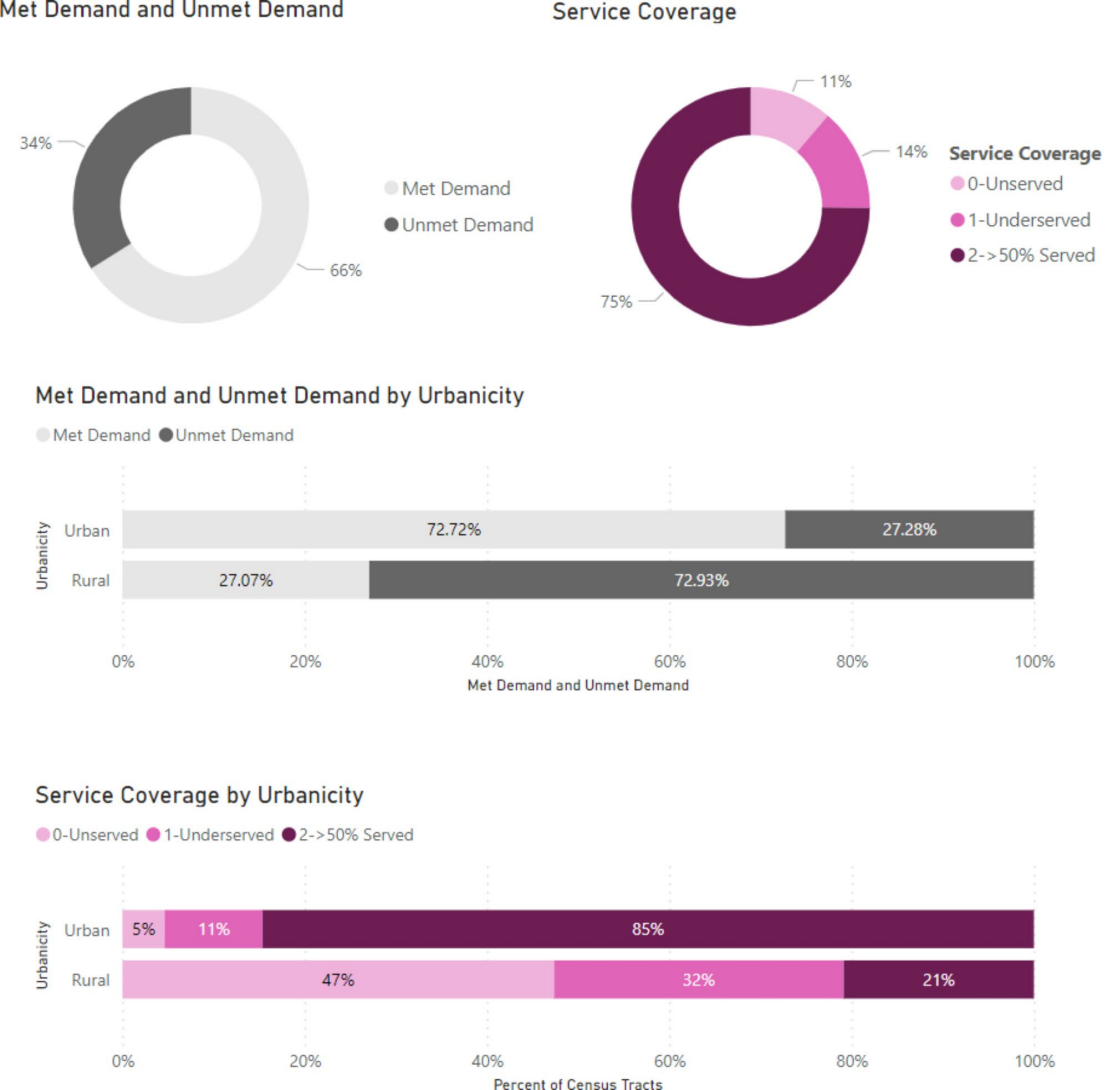
Baseline (no intervention) analysis of access measures (percent-met demand and service coverage) with Urbanicity

Figure 1 provides details on percent met demand and service coverage under baseline; 66% of the demand was met in 2018, with 73% and 27% met demand in urban and rural tracts, respectively. Among census tracts, 11% and 14% remained unserved and underserved, respectively; 15% and 79% were unserved/underserved urban and rural tracts, respectively.

### Intervention analysis

We compared the access outcomes based on (1) *direction* defined by the increase or decrease of the trend, (2) *difference magnitude* defined by the relative difference from the baseline, and (3) *position* defined by the position below/above/similar relative to the baseline.

**Fig. 2:**
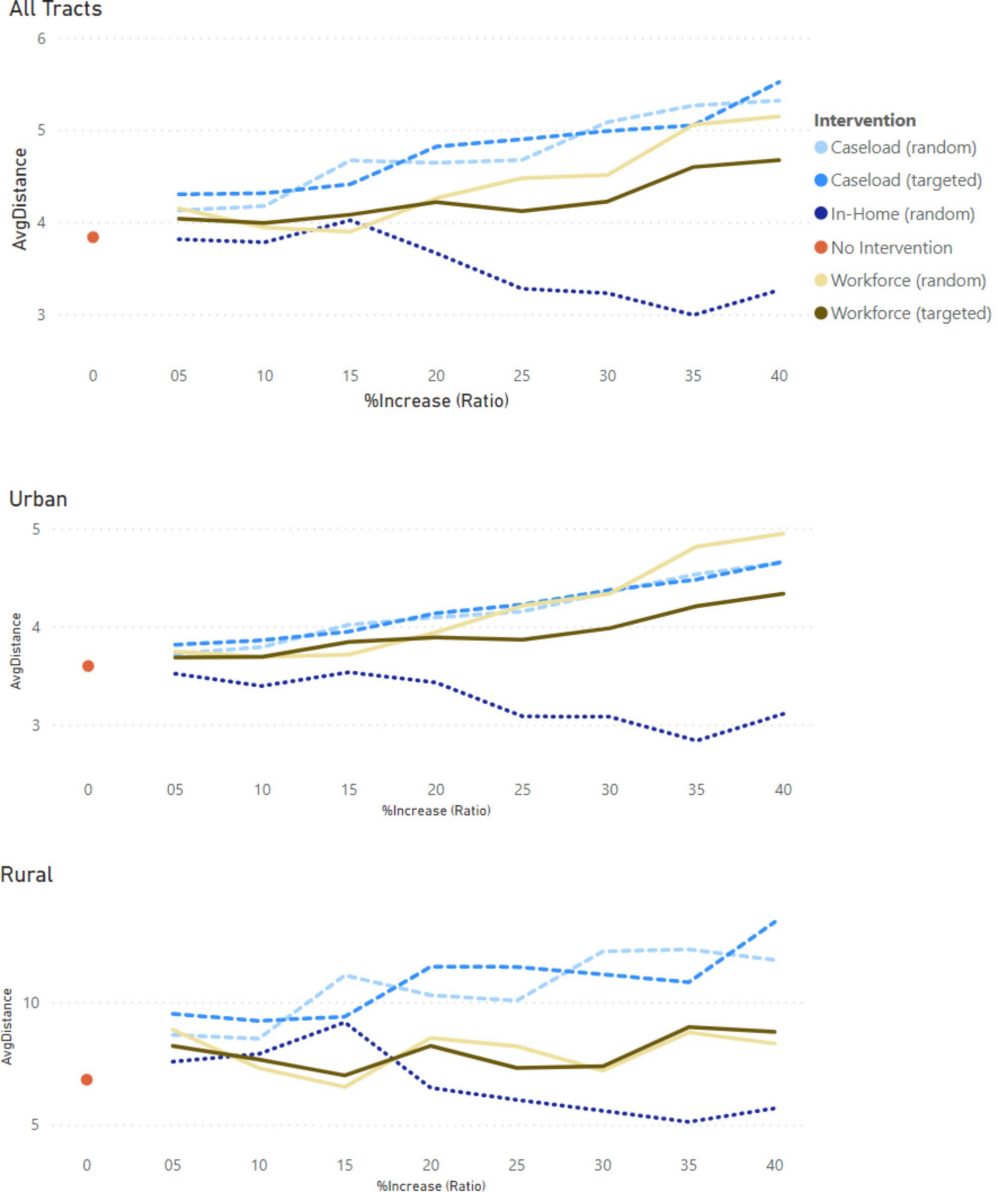
Trends of **travel distance** per in-clinic visit (miles) with increasing (5–40%) service caseload (caseload intervention: dashed lines), Medicaid participation (workforce intervention: solid lines), or shift of care from in-clinic to in-home (in-home intervention: dotted lines) across all census tracts (top), urban tracts (middle), and rural tracts (bottom) in Georgia. Solid dots represent baseline (no intervention)

**Fig. 3:**
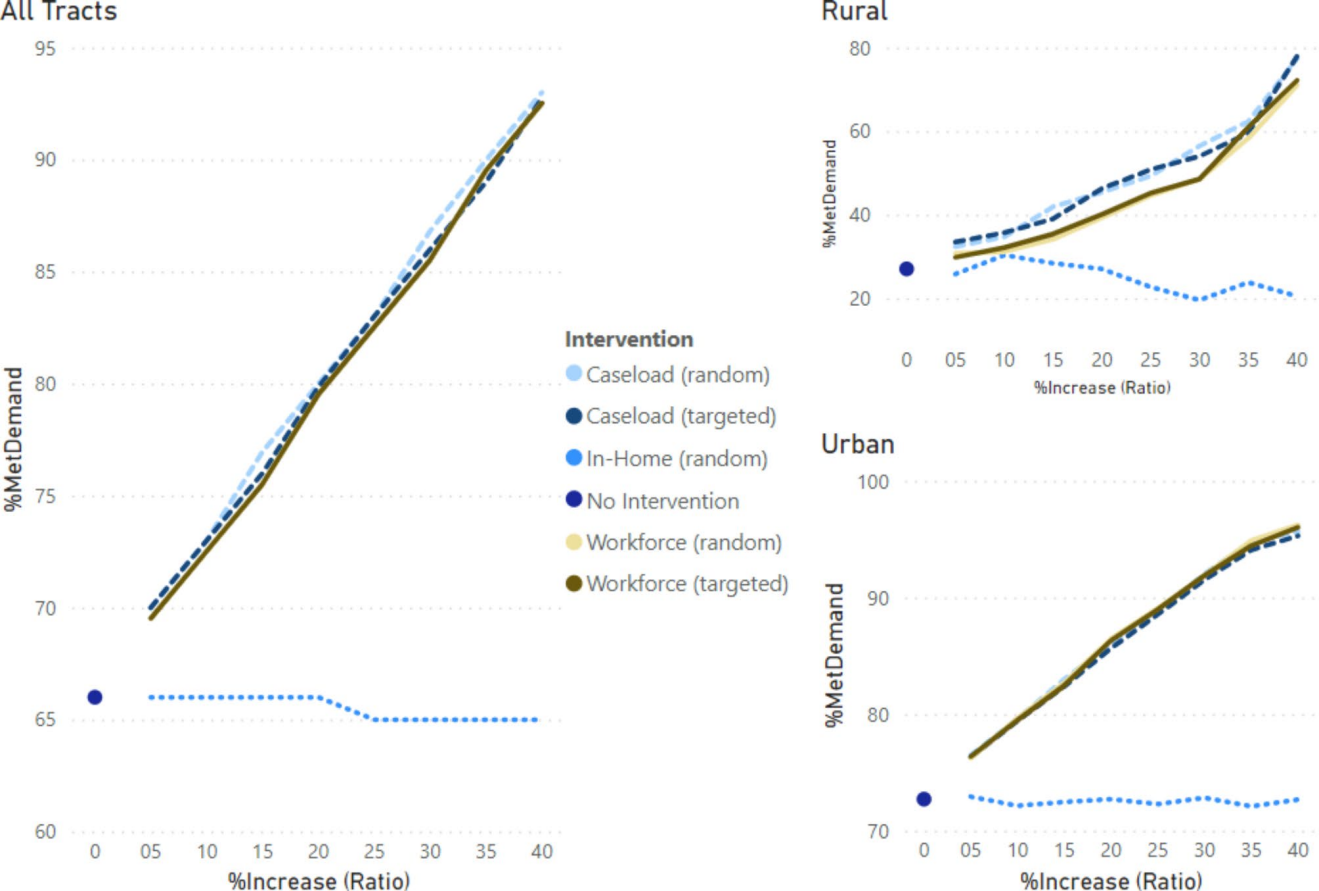
Trends of **percent met demand** with increasing (5–40%) service caseload (caseload intervention: dashed lines), in Medicaid participation (workforce intervention: solid lines), or shift of care from in-clinic to in-home (in-home intervention: dotted lines) across all census tracts (left figure), rural tracts (upper-right figure) and urban tracts (lower-right figure) in Georgia. Solid dots represent baseline (no intervention)

**Fig. 4:**
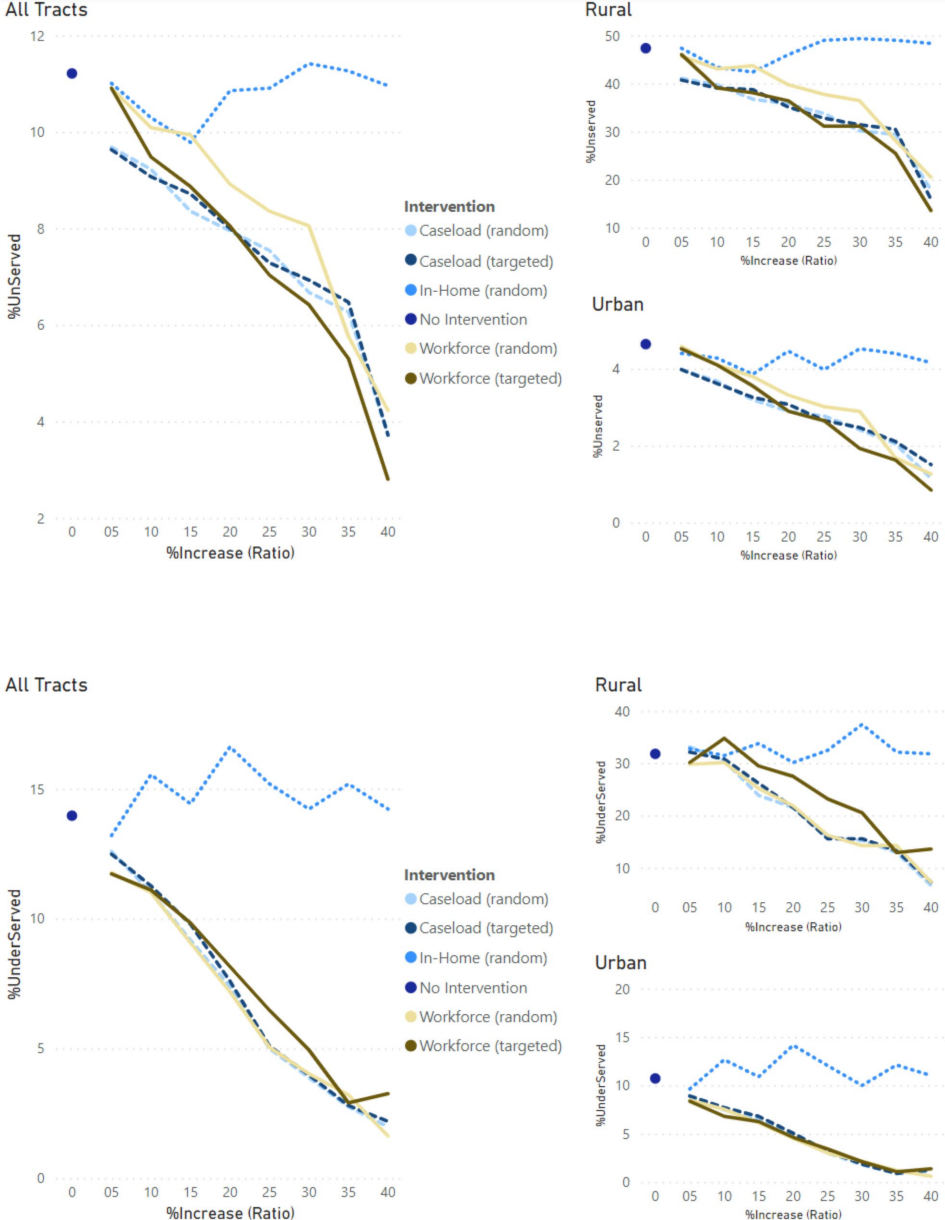
Trends of **service coverage** as percent of unserved (top) and underserved (bottom) communities with increasing (5–40%) service caseload (caseload intervention: dashed lines), Medicaid participation (workforce intervention: solid lines, ) or shift of care from in-clinic to in-home (in-home intervention: dotted liens) across all census tracts (left), rural tracts (upper-right), and urban tracts (lower-right) in Georgia. Solid dots represent baseline (no intervention)

Figures 2, 3 and 4 provide the trends by varying change or increase in ratios (x-axis) for the interventions versus baseline for the three outcome measures (travel distance, percent met-demand and service coverage). These figures are accompanied by numeric summaries for all tracts in Table 1 and by rurality-urbanicity in Table C4.

#### Travel distance outcome

In-home intervention was the only scenario with a decreasing trend, implying reduced travel distance for accessing psychosocial services (from 3.84 to 2.99 miles at 35% ratio). This trend was consistent across rural and urban tracts.

The other scenarios had an increasing trend, positioned above the baseline (from 5 to 5.5 miles at a 40% ratio). For urban tracts and a 40% ration, the caseload interventions increased to 4.65 miles, higher than the workforce-targeted scenario (4.33 miles) but lower than the workforce-random scenario (4.99 miles). For rural tracts and a 40% ratio, caseload interventions increased (~ 12 miles) more than workforce interventions (~ 8 miles). Results were consistent for other ratios.

#### Percent-met demand outcome

Under in-home intervention, the percent-met demand remained similar to the baseline.

Under caseload and workforce interventions, the percent-met demand had a linearly increasing trend above baseline, from 65% met-demand (baseline) to 93% met-demand (40% ratio change). Both interventions showed similar magnitude and similar trends. These results were consistent for urban tracts. For rural tracts, caseload intervention had a larger change, from 27 to 77% (40% ratio) compared to workforce intervention, from 27 to 72% (40% ratio). Results were similar for other ratios.

#### Service coverage outcome

In-home intervention did not change the percentage of unserved and underserved census tracts compared to the baseline. Caseload and workforce interventions both showed decreasing trends and lower trends versus baseline.

Caseload interventions decreased the percentage of unserved tracts from 10% (5% ratio) to 4% (40% ratio).

Workforce intervention decreased the percentage of unserved tracts from 11% (5% ratio) to 4% (40% ratio) under the random approach and 3% under the targeted approach, outperforming the other scenarios. For rural unserved tracts, the percentage decreased from 47% (baseline) to 14% compared to 16% (caseload, targeted), 18% (caseload, random), and 21% (workforce, random).

Both caseload and workforce interventions decreased the percentage of underserved tracts from ~ 13% to ~ 2%. For urban underserved tracts, all four scenarios looked identical, decreasing from 11% (baseline) to 1% (40% ratio). For rural underserved tracts, caseload interventions decreased the percentage from 32% (5% ratio) to 7% (40% ratio) whereas workforce interventions decreased to 7% (40% ratio, random) and 14% (40% ratio, targeted).

## Discussion

This study derived inferences on access to psychosocial services for Medicaid-insured children in Georgia. The access barrier of interest was the availability of MH-specialized providers while accounting for other access barriers identified in the literature [10], including accessibility through optimizing travel distance to receive care and accommodation through the consideration of different care settings. The study considered the complexities of the MH care system when evaluating access to psychosocial services, where providers have different specializations, delivering care in individual practices or organizations under multiple care settings. Evaluating access to psychosocial services needs to consider all such complexities because of the multiple facets of healthcare access [11].

The principal finding was that 34% of the demand for psychosocial services remained unmet overall. The unmet demand was much higher in rural tracts compared to urban tracts, with 79% of the rural Georgia identified being unserved, *establishing lack of adequacy of the MH care network to deliver psychosocial services to Medicaid-insured children*,* notably in rural areas*. The main source of inadequacy was limited availability, coming both from the limited caseload of existing MH providers and low Medicaid participation, rather than travel constraints. Travel distance was low for in-clinic visits, also with a large percentage of services delivered in-home or in-school care settings.

The extensive unmet demand was primarily due to the Medicaid participation rate, with a rate of 10% for MH organization providers and 14% for individual providers. MH organizations/centers delivered most of the psychosocial services (63%) with about 35% of psychosocial services were delivered outside the clinic settings (in-home or in-school) and primarily by MH organizations/centers. It is common for MH organizations to participate in Medicaid programs, since they can accommodate multiple licensure requirements and can submit to extensive Medicaid credentialing, offering opportunities for supervision and training, while also having the ability to secure Medicaid reimbursement and credentials.

To further understand the shortage in psychosocial service availability for Medicaid, we evaluated ‘what-if’ provider availability interventions. The first intervention assumed reallocating services to be delivered from in-clinic to in-home, overcoming travel barriers. More than 90% of in-home services were delivered by MH organizations, hence primarily targeted for this intervention. However, this intervention brought little to no improvement in reducing unmet demand. Other interventions directly targeted availability of services to Medicaid-insured children, increasing caseloads of Medicaid-participating providers or increasing Medicaid participation. Comparing the two interventions, their overall improvement was similar, with increasing participation slightly better at reducing the number of unserved communities. However, since increasing participation did not necessarily imply that the added providers would allocate sufficient caseload for Medicaid-insured children, increasing caseloads of existing providers may then be a more effective intervention since existing Medicaid-participating providers already had a sufficient network coverage geographically.

Our findings must be considered within the context of study limitations. This study was conducted using multiple data sets, including an administrative claims database. Claims data may not fully capture MH diagnosis and treatment since these data were designed for billing purposes [22]. Misdiagnosis could reduce the accuracy of the estimates on demand and caseload. We used the 2018 Medicaid data for characterizing in-home and in-clinic delivery of psychosocial services; except for the expansion of telehealth services in the COVID years, no major policy had been implemented in the sequel years that could have impacted a significant change in the results from those presented in this paper. In fact, using 2020–2021 data may have biased our results due to the impact of COVID-19 policies. We have also used the NPPES database to locate MH providers and practices along with their specialization, but the NPPES data only includes providers who can be reimbursed for their services. However, psychosocial services could be delivered by licensed and non-licensed providers or/and with different scopes of practice, easier to be reimbursed under MH organizations. We attempted to address this limitation by considering both individual and organization providers, with their caseload estimated using the Medicaid claims data.

We used data from a single state, and these findings may not necessarily generalize to other states. It is worth noting, however, that Georgia is a large state with a racially and ethnically diverse population, and a high percentage of children living in poverty [23]. Georgia has also promoted delivery of care under all three care settings, with recent policies supporting delivery of in-school care [16].

Notwithstanding limitations, our study provided a first granular examination of access to psychosocial services for Medicaid-insured children in Georgia. This study highlighted the importance of the provision of psychosocial services under all three care settings (in-clinic, in-home, in-school), making those services accessible, with reasonable travel distances overall, and accommodating children’s needs and their parents’ efforts towards travel. This finding concurs with current investments in community-based care [24, 25].

Despite policy efforts, the availability of MH providers and their levels of Medicaid participation deemed the network of providers inadequate for Medicaid-insured children in Georgia. Community MH organizations face challenges regarding workforce recruitment and retention [26, 27] due to large differential between private and public compensation, additional efforts required to deliver community-based services, extensive requirements for Medicaid credentialing in addition to licensing and certification among others. These challenges could be addressed by streamlining the Medicaid participation requirements and reimbursement fee schedules to be on-par with other medical services.

## Conclusion

We used optimization-based access modeling to evaluate the access to psychosocial services for Medicaid-insured children in Georgia. Through investigating multiple intervention scenarios, we found that the main source of network inadequacy was the scarcity of MH providers available to Medicaid-insured children rather than travel constraints. Provider inadequacy arose from both the limited caseload of existing MH providers and low Medicaid participation, and we concluded that either increasing provider caseload or expanding Medicaid participation could reduce unmet demand, thus improving access. Among these two interventions, we found that increasing caseloads were the most effective as existing Medicaid-participating providers already had sufficient network coverage geographically.

## Electronic supplementary material

Below is the link to the electronic supplementary material.

Supplementary Material 1

Supplementary Material 2

Supplementary Material 3

## Data Availability

The datasets used and/or analyzed during the current study are available from the corresponding author on reasonable request.

## Abbreviations

CMS: Centers of Medicare and Medicaid Services
CPT: Current Procedural Terminology
DBHDD: Department of Behavioral Health and Developmental Disabilities
HCPCS: Healthcare Common Procedure Coding System
ICD-10: International Classification of Diseases, 10th Revision
MH: Mental Health
MHE1: Mental Health Entity 1
MHE2: Mental Health Entity 2
NPPES: National Plan and Provider Enumeration System
QNA: Quantitative Network Adequacy
RUCA: Rural-Urban Commuting Area
T-MSIS: Transformed Medicaid Statistical Information System
TAF: T-MSIS Analytic Files
