# Supplementary material for: Evaluating access to psychosocial services for the medicaid-insured children in Georgia
Source: BMC Public Health. 2025 Jan 20;25:244. doi: 10.1186/s12889-025-21374-7 (PMC11748264; doi:10.1186/s12889-025-21374-7)
Supplement: Supplementary file 1 — Supplementary Material 1 [file 12889_2025_21374_MOESM1_ESM.docx]

Online-Supplement A

This online supplement includes details on the derivation of the demand and supply for Mental Health (MH) services. The following supplemental material is included in this supplement.

Table of Contents

[11-cell Rule 2](#_Toc176883432)

[Figure A1. Procedure for Imputing Missing Data due to 11-cell Rule 2](#_Toc176883433)

[Figure A2. Flowchart of Census-Tract Demand Derivation 3](#_Toc176883434)

[Procedure for Deriving Practice-Level MH Supply and Corresponding Accessibility 4](#_Toc176883435)

[Figure A3. Flowchart of Practice-Level MH Supply Derivation 6](#_Toc176883436)

[Figure A4. Flowchart of Service Type Classification 6](#_Toc176883437)

[Figure A5. Procedure to Match NPI with Practice Address 7](#_Toc176883438)

[Figure A6. Procedure to Estimate In-School Assignments 8](#_Toc176883439)

[Figure A7. Procedure to Determine In-home Coverage 8](#_Toc176883440)

[Additional Classifications 10](#_Toc176883441)

[Tables 11](#_Toc176883442)

[Table A1. MH ICD-10 Diagnosis Codes 11](#_Toc176883443)

[Table A2. Psychotherapy Procedure Codes (CPT Codes) 17](#_Toc176883444)

[Table A3. Census Tract Urbanicity Classification 20](#_Toc176883445)

[Table A4. Taxonomy Code Categorization 21](#_Toc176883446)

[References 22](#_Toc176883447)

## 11-cell Rule

Compliant to HIPPA, we removed any patient-identifiable information in reporting. That is, for demand, we masked the number of demanded visits and the number of child patients on census tracts with fewer than 11 child patients. Similarly for supply, for each provider under each type of services (i.e., in-clinic, in-home), we masked their number of caseloads if they served fewer than 11 child patients. The detailed procedure is illustrated in Figure A1.

### Figure A1. Procedure for Imputing Missing Data due to 11-cell Rule


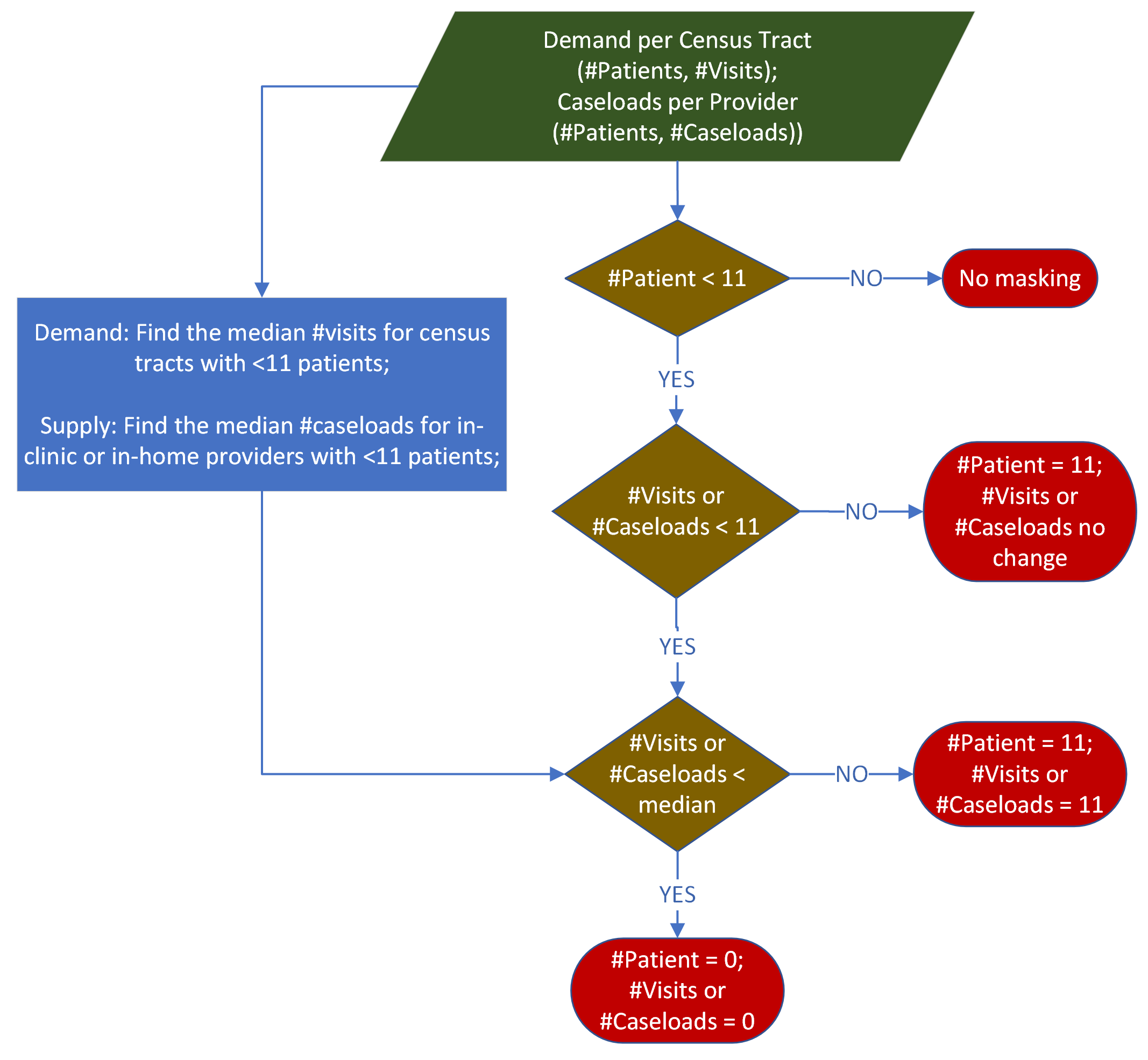


### Figure A2. Flowchart of Census-Tract Demand Derivation


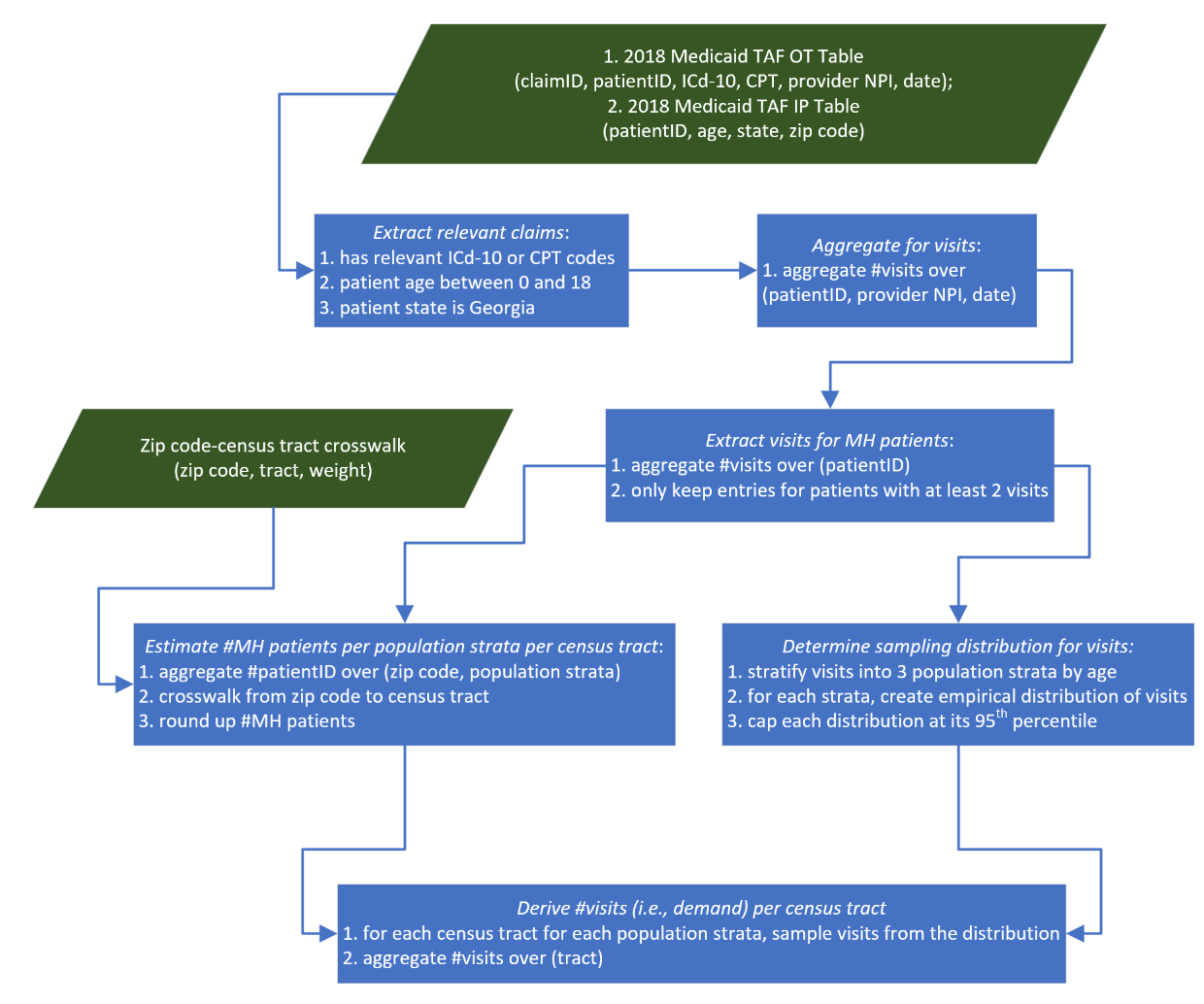


## Procedure for Deriving Practice-Level MH Supply and Corresponding Accessibility

We consider psychosocial services provided under three settings (i.e., type of service): in-clinic, in-home, and in-school. Realized supply of psychosocial services provided at each practice location (i.e., practice-level) was measured as caseloads, which was the aggregation of number of visits to practice addresses. For in-school caseloads, we used data from 2019 APEX dataset to estimate the assignment between each school address (i.e., practice location) and each nearby census tracts:

1. Computing the yearly caseloads at each APEX-participating schools
2. Locating the nearby census tracts from each school addresses
3. Distributing the number of caseloads from each school to its nearby census tracts, weighted by census tract population.

Figure A2 illustrated the overall flow for estimating assignment of in-school services.

For in-clinic and in-home supply, we applied a three-step procedure to estimate their caseloads from 2018 Medicaid claims:

1. Identifying claims with relevant CPT codes, serving Georgia children aged 18 and below, under in-clinic or in-home service types.
2. Aggregating the number of visits first for each provider NPI, differentiating the visits from in-clinic and in-home providers
3. Cross-referencing the practice location for provider NPIs, then aggregating the number of visits per practice location per service type

Figure A3 illustrated the overall flow for deriving practice-level MH supply.

In step one, the list of CPT codes relevant to psychosocial services was provided in Table A2, and this list was derived using the list of MH ICD-10 diagnosis coded in Table A1. Based on claims with MH ICD-10 codes, we drew a frequency table of the corresponding CPT codes, the procedure codes used to identify psychosocial services. Specifically, we considered all the CPT codes corresponding to the claims with MH diagnoses for children aged 3-18 and their frequency across all those claims. We selected those codes with the highest frequency, making up 95% of the claims. These CPT codes were then reviewed by clinicians at Georgia HOPE led by Nikki Raymond, and only relevant codes were included. This approach was applied to the 2018 national Medicaid claims data hence relevant not only to Georgia. The service type associated with each claim was identified with Figure A4.

In step three, we aggregated the number of visits at each practice location. Figure A5 illustrated how we identified the practice location associated with each provider NPI using POS_CD and TOS_CD from Medicaid claims. Relevant POS_CD and TOS_CD codes were provided in Table A4.

We further restricted the accessibility to practice locations from census tracts under realistic considerations. For in-clinic services, we capped the maximum travel distance between census tract and provider location. For in-home services, we restricted the list of counties that each in-home provider could serve and cover, illustrated as Figure A6.

### Figure A3. Flowchart of Practice-Level MH Supply Derivation


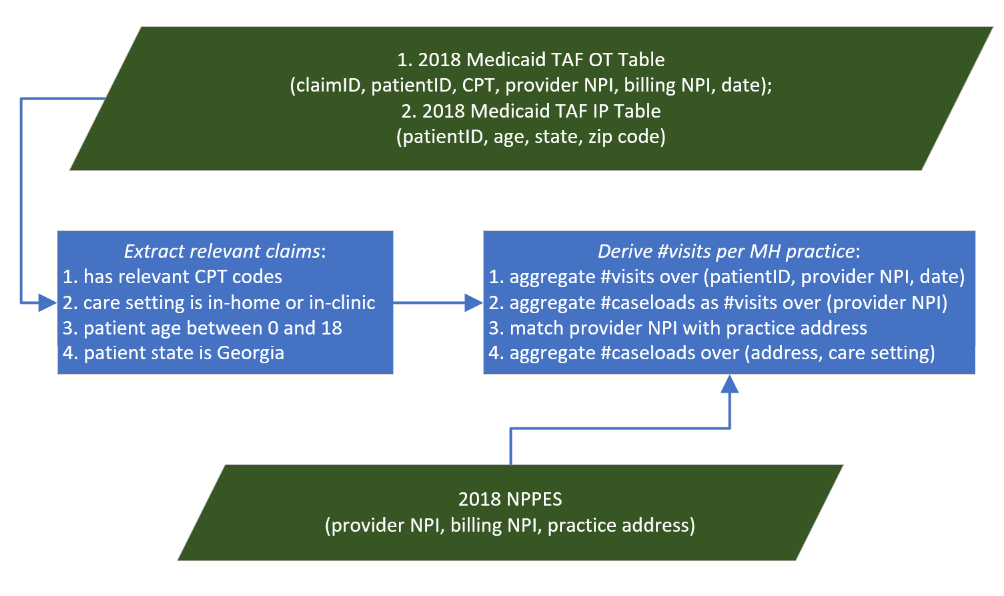


### Figure A4. Flowchart of Service Type Classification

This flowchart summarized the procedure to classify a claim as either in-home or in-clinic claim, which will be subsequently used to compute the caseload under in-home/in-clinic care settings. We use both the place of service (POS_CD) codes and type of service (TOS_CD) codes for classification.


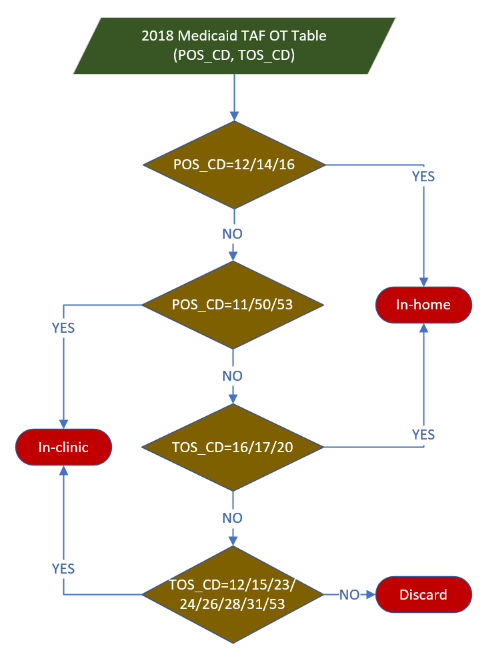


### Figure A5. Procedure to Match NPI with Practice Address


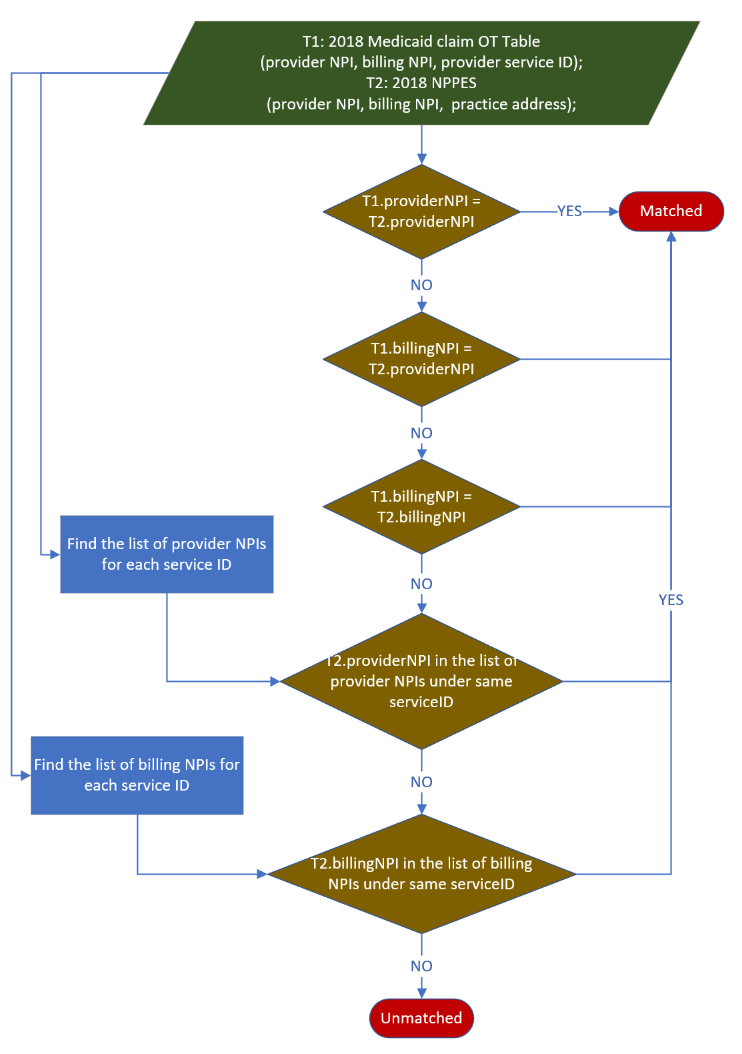


### Figure A6. Procedure to Estimate In-School Assignments


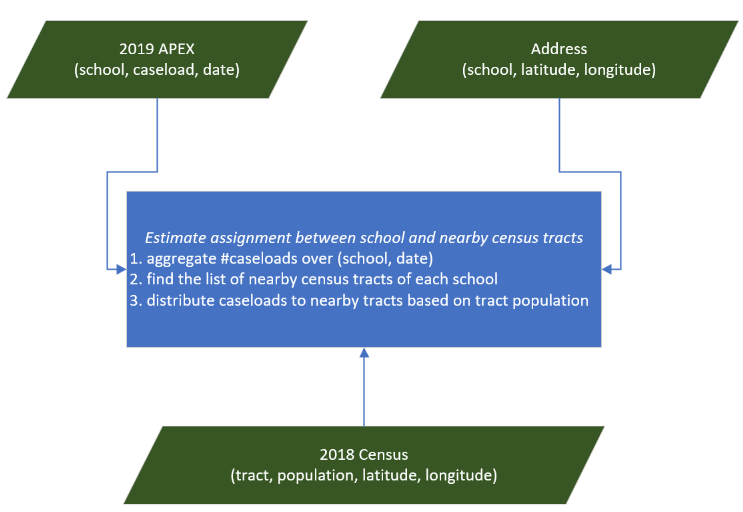


### Figure A7. Procedure to Determine In-home Coverage


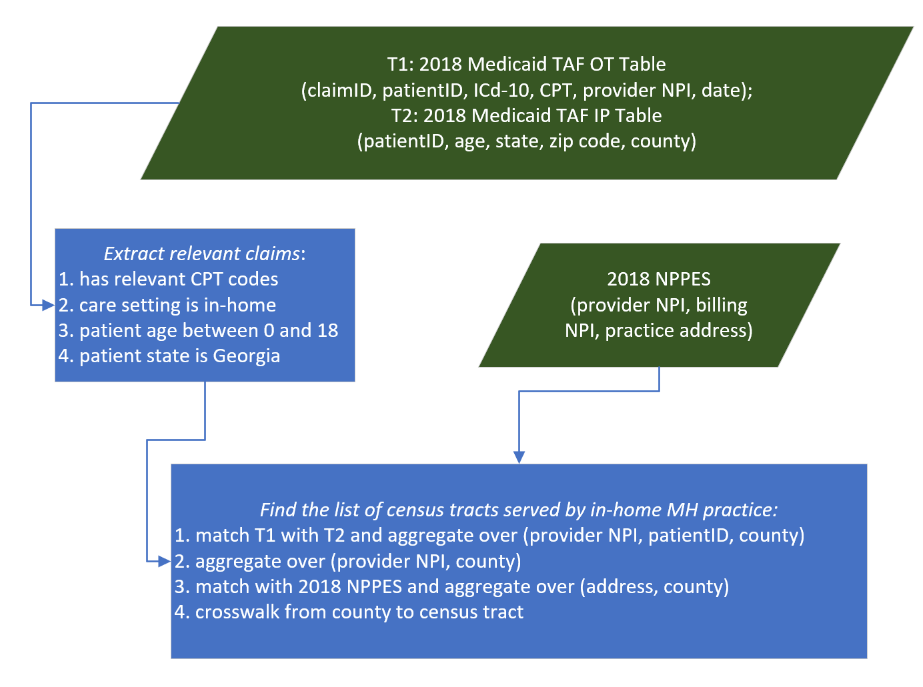


## Additional Classifications

To assist demand and supply analysis, we further classified census tracts into either urban tracts or rural tracts based on Rural-Urban Continuum Code (RUCC) [2]. The exact breakdown was provided in Table A3. We also classified each provider NPI by their primary taxonomy [3], detailed in Table A4.

## Tables

### Table A1. MH ICD-10 Diagnosis Codes

| Category | ICD10 | Description |
| --- | --- | --- |
| ADHD | F90 | Attention-deficit hyperactivity disorders |
| ADHD | F900 | Attn-defct hyperactivity disorder, predom inattentive type |
| ADHD | F901 | Attn-defct hyperactivity disorder, predom hyperactive type |
| ADHD | F902 | Attention-deficit hyperactivity disorder, combined type |
| ADHD | F908 | Attention-deficit hyperactivity disorder, other type |
| ADHD | F909 | Attention-deficit hyperactivity disorder, unspecified type |
| Adjustment Reaction | F43 | Reaction to severe stress, and adjustment disorders |
| Adjustment Reaction | F430 | Acute stress reaction |
| Adjustment Reaction | F431 | Post-traumatic stress disorder (ptsd) |
| Adjustment Reaction | F4310 | Post-traumatic stress disorder, unspecified |
| Adjustment Reaction | F4311 | Post-traumatic stress disorder, acute |
| Adjustment Reaction | F4312 | Post-traumatic stress disorder, chronic |
| Adjustment Reaction | F432 | Adjustment disorders |
| Adjustment Reaction | F4320 | Adjustment disorder, unspecified |
| Adjustment Reaction | F4321 | Adjustment disorder with depressed mood |
| Adjustment Reaction | F4322 | Adjustment disorder with anxiety |
| Adjustment Reaction | F4323 | Adjustment disorder with mixed anxiety and depressed mood |
| Adjustment Reaction | F4324 | Adjustment disorder with disturbance of conduct |
| Adjustment Reaction | F4325 | Adjustment disorder w mixed disturb of emotions and conduct |
| Adjustment Reaction | F4329 | Adjustment disorder with other symptoms |
| Adjustment Reaction | F438 | Other reactions to severe stress |
| Adjustment Reaction | F439 | Reaction to severe stress, unspecified |
| Adjustment Reaction | F93 | Emotional disorders with onset specific to childhood |
| Adjustment Reaction | F930 | Separation anxiety disorder of childhood |
| Adjustment Reaction | F938 | Other childhood emotional disorders |
| Adjustment Reaction | F939 | Childhood emotional disorder, unspecified |
| Adjustment Reaction | F94 | Disord social w onset specific to childhood and adolescence |
| Adjustment Reaction | F940 | Selective mutism |
| Adjustment Reaction | F941 | Reactive attachment disorder of childhood |
| Adjustment Reaction | F942 | Disinhibited attachment disorder of childhood |
| Adjustment Reaction | F948 | Other childhood disorders of social functioning |
| Adjustment Reaction | F949 | Childhood disorder of social functioning, unspecified |
| Anxiety | F40 | Phobic anxiety disorders |
| Anxiety | F400 | Agoraphobia |
| Anxiety | F4000 | Agoraphobia, unspecified |
| Anxiety | F4001 | Agoraphobia with panic disorder |
| Anxiety | F4002 | Agoraphobia without panic disorder |
| Anxiety | F401 | Social phobias |
| Anxiety | F4010 | Social phobia, unspecified |
| Anxiety | F4011 | Social phobia, generalized |
| Anxiety | F402 | Specific (isolated) phobias |
| Anxiety | F4021 | Animal type phobia |
| Anxiety | F40210 | Arachnophobia |
| Anxiety | F40218 | Other animal type phobia |
| Anxiety | F4022 | Natural environment type phobia |
| Anxiety | F40220 | Fear of thunderstorms |
| Anxiety | F40228 | Other natural environment type phobia |
| Anxiety | F4023 | Blood, injection, injury type phobia |
| Anxiety | F40230 | Fear of blood |
| Anxiety | F40231 | Fear of injections and transfusions |
| Anxiety | F40232 | Fear of other medical care |
| Anxiety | F40233 | Fear of injury |
| Anxiety | F4024 | Situational type phobia |
| Anxiety | F40240 | Claustrophobia |
| Anxiety | F40241 | Acrophobia |
| Anxiety | F40242 | Fear of bridges |
| Anxiety | F40243 | Fear of flying |
| Anxiety | F40248 | Other situational type phobia |
| Anxiety | F4029 | Other specified phobia |
| Anxiety | F40290 | Androphobia |
| Anxiety | F40291 | Gynephobia |
| Anxiety | F40298 | Other specified phobia |
| Anxiety | F408 | Other phobic anxiety disorders |
| Anxiety | F409 | Phobic anxiety disorder, unspecified |
| Anxiety | F41 | Other anxiety disorders |
| Anxiety | F410 | Panic disorder [episodic paroxysmal anxiety] |
| Anxiety | F411 | Generalized anxiety disorder |
| Anxiety | F413 | Other mixed anxiety disorders |
| Anxiety | F418 | Other specified anxiety disorders |
| Anxiety | F419 | Anxiety disorder, unspecified |
| Anxiety | F42 | Obsessive-compulsive disorder |
| Anxiety | F422 | Mixed obsessional thoughts and acts |
| Anxiety | F423 | Hoarding disorder |
| Anxiety | F424 | Excoriation (skin-picking) disorder |
| Anxiety | F428 | Other obsessive-compulsive disorder |
| Anxiety | F429 | Obsessive-compulsive disorder, unspecified |
| Anxiety | F43 | Reaction to severe stress, and adjustment disorders |
| Anxiety | F430 | Acute stress reaction |
| Anxiety | F431 | Post-traumatic stress disorder (ptsd) |
| Anxiety | F4310 | Post-traumatic stress disorder, unspecified |
| Anxiety | F4311 | Post-traumatic stress disorder, acute |
| Anxiety | F4312 | Post-traumatic stress disorder, chronic |
| Anxiety | F432 | Adjustment disorders |
| Anxiety | F4320 | Adjustment disorder, unspecified |
| Anxiety | F4321 | Adjustment disorder with depressed mood |
| Anxiety | F4322 | Adjustment disorder with anxiety |
| Anxiety | F4323 | Adjustment disorder with mixed anxiety and depressed mood |
| Anxiety | F4324 | Adjustment disorder with disturbance of conduct |
| Anxiety | F4325 | Adjustment disorder w mixed disturb of emotions and conduct |
| Anxiety | F4329 | Adjustment disorder with other symptoms |
| Anxiety | F438 | Other reactions to severe stress |
| Anxiety | F439 | Reaction to severe stress, unspecified |
| Anxiety | F93 | Emotional disorders with onset specific to childhood |
| Anxiety | F930 | Separation anxiety disorder of childhood |
| Anxiety | F938 | Other childhood emotional disorders |
| Anxiety | F939 | Childhood emotional disorder, unspecified |
| Anxiety | R45 | Symptoms and signs involving emotional state |
| Anxiety | R450 | Nervousness |
| Anxiety | R451 | Restlessness and agitation |
| Anxiety | R452 | Unhappiness |
| Anxiety | R453 | Demoralization and apathy |
| Anxiety | R454 | Irritability and anger |
| Anxiety | R455 | Hostility |
| Anxiety | R456 | Violent behavior |
| Anxiety | R457 | State of emotional shock and stress, unspecified |
| Anxiety | R458 | Other symptoms and signs involving emotional state |
| Anxiety | R4581 | Low self-esteem |
| Anxiety | R4582 | Worries |
| Anxiety | R4583 | Excessive crying of child, adolescent or adult |
| Anxiety | R4584 | Anhedonia |
| Anxiety | R4585 | Homicidal and suicidal ideations |
| Anxiety | R45850 | Homicidal ideations |
| Anxiety | R45851 | Suicidal ideations |
| Anxiety | R4586 | Emotional lability |
| Anxiety | R4587 | Impulsiveness |
| Anxiety | R4589 | Other symptoms and signs involving emotional state |
| Conduct Disorder | F63 | Impulse disorders |
| Conduct Disorder | F630 | Pathological gambling |
| Conduct Disorder | F631 | Pyromania |
| Conduct Disorder | F632 | Kleptomania |
| Conduct Disorder | F633 | Trichotillomania |
| Conduct Disorder | F638 | Other impulse disorders |
| Conduct Disorder | F6381 | Intermittent explosive disorder |
| Conduct Disorder | F6389 | Other impulse disorders |
| Conduct Disorder | F639 | Impulse disorder, unspecified |
| Conduct Disorder | F91 | Conduct disorders |
| Conduct Disorder | F910 | Conduct disorder confined to family context |
| Conduct Disorder | F911 | Conduct disorder, childhood-onset type |
| Conduct Disorder | F912 | Conduct disorder, adolescent-onset type |
| Conduct Disorder | F913 | Oppositional defiant disorder |
| Conduct Disorder | F918 | Other conduct disorders |
| Conduct Disorder | F919 | Conduct disorder, unspecified |
| Depressive Disorder | F32 | Major depressive disorder, single episode |
| Depressive Disorder | F320 | Major depressive disorder, single episode, mild |
| Depressive Disorder | F321 | Major depressive disorder, single episode, moderate |
| Depressive Disorder | F322 | Major depressv disord, single epsd, sev w/o psych features |
| Depressive Disorder | F323 | Major depressv disord, single epsd, severe w psych features |
| Depressive Disorder | F324 | Major depressv disorder, single episode, in partial remis |
| Depressive Disorder | F325 | Major depressive disorder, single episode, in full remission |
| Depressive Disorder | F328 | Other depressive episodes |
| Depressive Disorder | F3281 | Premenstrual dysphoric disorder |
| Depressive Disorder | F3289 | Other specified depressive episodes |
| Depressive Disorder | F329 | Major depressive disorder, single episode, unspecified |
| Depressive Disorder | F33 | Major depressive disorder, recurrent |
| Depressive Disorder | F330 | Major depressive disorder, recurrent, mild |
| Depressive Disorder | F331 | Major depressive disorder, recurrent, moderate |
| Depressive Disorder | F332 | Major depressv disorder, recurrent severe w/o psych features |
| Depressive Disorder | F333 | Major depressv disorder, recurrent, severe w psych symptoms |
| Depressive Disorder | F334 | Major depressive disorder, recurrent, in remission |
| Depressive Disorder | F3340 | Major depressive disorder, recurrent, in remission, unsp |
| Depressive Disorder | F3341 | Major depressive disorder, recurrent, in partial remission |
| Depressive Disorder | F3342 | Major depressive disorder, recurrent, in full remission |
| Depressive Disorder | F338 | Other recurrent depressive disorders |
| Depressive Disorder | F339 | Major depressive disorder, recurrent, unspecified |
| Depressive Disorder | F34 | Persistent mood [affective] disorders |
| Depressive Disorder | F340 | Cyclothymic disorder |
| Depressive Disorder | F341 | Dysthymic disorder |
| Depressive Disorder | F348 | Other persistent mood [affective] disorders |
| Depressive Disorder | F3481 | Disruptive mood dysregulation disorder |
| Depressive Disorder | F3489 | Other specified persistent mood disorders |
| Depressive Disorder | F349 | Persistent mood [affective] disorder, unspecified |
| Depressive Disorder | F43 | Reaction to severe stress, and adjustment disorders |
| Depressive Disorder | F430 | Acute stress reaction |
| Depressive Disorder | F431 | Post-traumatic stress disorder (ptsd) |
| Depressive Disorder | F4310 | Post-traumatic stress disorder, unspecified |
| Depressive Disorder | F4311 | Post-traumatic stress disorder, acute |
| Depressive Disorder | F4312 | Post-traumatic stress disorder, chronic |
| Depressive Disorder | F432 | Adjustment disorders |
| Depressive Disorder | F4320 | Adjustment disorder, unspecified |
| Depressive Disorder | F4321 | Adjustment disorder with depressed mood |
| Depressive Disorder | F4322 | Adjustment disorder with anxiety |
| Depressive Disorder | F4323 | Adjustment disorder with mixed anxiety and depressed mood |
| Depressive Disorder | F4324 | Adjustment disorder with disturbance of conduct |
| Depressive Disorder | F4325 | Adjustment disorder w mixed disturb of emotions and conduct |
| Depressive Disorder | F4329 | Adjustment disorder with other symptoms |
| Depressive Disorder | F438 | Other reactions to severe stress |
| Depressive Disorder | F439 | Reaction to severe stress, unspecified |
| Other Mental Health Disorders (from Chapter 5) | F063 | Mood disorder due to known physiological condition |
| Other Mental Health Disorders (from Chapter 5) | F0630 | Mood disorder due to known physiological condition, unsp |
| Other Mental Health Disorders (from Chapter 5) | F0631 | Mood disorder due to known physiol cond w depressv features |
| Other Mental Health Disorders (from Chapter 5) | F0632 | Mood disord d/t physiol cond w major depressive-like epsd |
| Other Mental Health Disorders (from Chapter 5) | F0633 | Mood disorder due to known physiol cond w manic features |
| Other Mental Health Disorders (from Chapter 5) | F0634 | Mood disorder due to known physiol cond w mixed features |
| Other Mental Health Disorders (from Chapter 5) | F064 | Anxiety disorder due to known physiological condition |
| Other Mental Health Disorders (from Chapter 5) | F32 | Major depressive disorder, single episode |
| Other Mental Health Disorders (from Chapter 5) | F320 | Major depressive disorder, single episode, mild |
| Other Mental Health Disorders (from Chapter 5) | F321 | Major depressive disorder, single episode, moderate |
| Other Mental Health Disorders (from Chapter 5) | F322 | Major depressv disord, single epsd, sev w/o psych features |
| Other Mental Health Disorders (from Chapter 5) | F323 | Major depressv disord, single epsd, severe w psych features |
| Other Mental Health Disorders (from Chapter 5) | F324 | Major depressv disorder, single episode, in partial remis |
| Other Mental Health Disorders (from Chapter 5) | F325 | Major depressive disorder, single episode, in full remission |
| Other Mental Health Disorders (from Chapter 5) | F328 | Other depressive episodes |
| Other Mental Health Disorders (from Chapter 5) | F3281 | Premenstrual dysphoric disorder |
| Other Mental Health Disorders (from Chapter 5) | F3289 | Other specified depressive episodes |
| Other Mental Health Disorders (from Chapter 5) | F329 | Major depressive disorder, single episode, unspecified |
| Other Mental Health Disorders (from Chapter 5) | F33 | Major depressive disorder, recurrent |
| Other Mental Health Disorders (from Chapter 5) | F330 | Major depressive disorder, recurrent, mild |
| Other Mental Health Disorders (from Chapter 5) | F331 | Major depressive disorder, recurrent, moderate |
| Other Mental Health Disorders (from Chapter 5) | F332 | Major depressv disorder, recurrent severe w/o psych features |
| Other Mental Health Disorders (from Chapter 5) | F333 | Major depressv disorder, recurrent, severe w psych symptoms |
| Other Mental Health Disorders (from Chapter 5) | F334 | Major depressive disorder, recurrent, in remission |
| Other Mental Health Disorders (from Chapter 5) | F3340 | Major depressive disorder, recurrent, in remission, unsp |
| Other Mental Health Disorders (from Chapter 5) | F3341 | Major depressive disorder, recurrent, in partial remission |
| Other Mental Health Disorders (from Chapter 5) | F3342 | Major depressive disorder, recurrent, in full remission |
| Other Mental Health Disorders (from Chapter 5) | F338 | Other recurrent depressive disorders |
| Other Mental Health Disorders (from Chapter 5) | F339 | Major depressive disorder, recurrent, unspecified |
| Other Mental Health Disorders (from Chapter 5) | F34 | Persistent mood [affective] disorders |
| Other Mental Health Disorders (from Chapter 5) | F340 | Cyclothymic disorder |
| Other Mental Health Disorders (from Chapter 5) | F341 | Dysthymic disorder |
| Other Mental Health Disorders (from Chapter 5) | F348 | Other persistent mood [affective] disorders |
| Other Mental Health Disorders (from Chapter 5) | F3481 | Disruptive mood dysregulation disorder |
| Other Mental Health Disorders (from Chapter 5) | F3489 | Other specified persistent mood disorders |
| Other Mental Health Disorders (from Chapter 5) | F349 | Persistent mood [affective] disorder, unspecified |
| Other Mental Health Disorders (from Chapter 5) | F39 | Unspecified mood [affective] disorder |
| Other Mental Health Disorders (from Chapter 5) | F91 | Conduct disorders |
| Other Mental Health Disorders (from Chapter 5) | F910 | Conduct disorder confined to family context |
| Other Mental Health Disorders (from Chapter 5) | F911 | Conduct disorder, childhood-onset type |
| Other Mental Health Disorders (from Chapter 5) | F912 | Conduct disorder, adolescent-onset type |
| Other Mental Health Disorders (from Chapter 5) | F913 | Oppositional defiant disorder |
| Other Mental Health Disorders (from Chapter 5) | F918 | Other conduct disorders |
| Other Mental Health Disorders (from Chapter 5) | F919 | Conduct disorder, unspecified |

### Table A2. Psychotherapy Procedure Codes (CPT Codes)

| Code | Description |
| --- | --- |
| 90804 | Individual psychotherapy, insight oriented, behavior modifying and/or supportive, in an office or outpatient facility, approximately 20 to 30 minutes face-to-face with the patient |
| 90805 | Individual psychotherapy, insight oriented, behavior modifying and/or supportive, in an office or outpatient facility, approximately 20 to 30 minutes face-to-face with the patient; with medical evaluation and management services |
| 90806 | Individual psychotherapy, insight oriented, behavior modifying and/or supportive, in an office or outpatient facility, approximately 45 to 50 minutes face-to-face with the patient |
| 90807 | Individual psychotherapy, insight oriented, behavior modifying and/or supportive, in an office or outpatient facility, approximately 45 to 50 minutes face-to-face with the patient; with medical evaluation and management services |
| 90808 | Individual psychotherapy, insight oriented, behavior modifying and/or supportive, in an office or outpatient facility, approximately 75 to 80 minutes face-to-face with the patient |
| 90809 | Individual psychotherapy, insight oriented, behavior modifying and/or supportive, in an office or outpatient facility, approximately 75 to 80 minutes face-to-face with the patient; with medical evaluation and management services |
| 90810 | Individual psychotherapy, interactive, using play equipment, physical devices, language interpreter, or other mechanisms of non-verbal communication, in an office or outpatient facility, approximately 20 to 30 minutes face-to-face with the patient; |
| 90811 | Individual psychotherapy, interactive, using play equipment, physical devices, language interpreter, or other mechanisms of non-verbal communication, in an office or outpatient facility, approximately 20 to 30 minutes face-to-face with the patient; with medical evaluation and management services |
| 90812 | Individual psychotherapy, interactive, using play equipment, physical devices, language interpreter, or other mechanisms of non-verbal communication, in an office or outpatient facility, approximately 45 to 50 minutes face-to-face with the patient; |
| 90813 | Individual psychotherapy, interactive, using play equipment, physical devices, language interpreter, or other mechanisms of non-verbal communication, in an office or outpatient facility, approximately 45 to 50 minutes face-to-face with the patient; with medical evaluation and management services |
| 90814 | Individual psychotherapy, interactive, using play equipment, physical devices, language interpreter, or other mechanisms of non-verbal communication, in an office or outpatient facility, approximately 75 to 80 minutes face-to-face with the patient; |
| 90815 | Individual psychotherapy, interactive, using play equipment, physical devices, language interpreter, or other mechanisms of non-verbal communication, in an office or outpatient facility, approximately 75 to 80 minutes face-to-face with the patient; with medical evaluation and management services |
| 90816 | Individual psychotherapy, insight oriented, behavior modifying and/or supportive, in an inpatient hospital, partial hospital or residential care setting, approximately 20 to 30 minutes face-to-face with the patient; |
| 90817 | Individual psychotherapy, insight oriented, behavior modifying and/or supportive, in an inpatient hospital, partial hospital or residential care setting, approximately 20 to 30 minutes face-to-face with the patient; with medical evaluation and management services |
| 90818 | Individual psychotherapy, insight oriented, behavior modifying and/or supportive, in an inpatient hospital, partial hospital or residential care setting, approximately 45 to 50 minutes face-to-face with the |
| 90819 | Individual psychotherapy, insight oriented, behavior modifying and/or supportive, in an inpatient hospital, partial hospital or residential care setting, approximately 45 to 50 minutes face-to-face with the patient; with medical evaluation and management services |
| 90821 | Individual psychotherapy, insight oriented, behavior modifying and/or supportive, in an inpatient hospital, partial hospital or residential care setting, approximately 75 to 80 minutes face-to-face with the patient |
| 90822 | Individual psychotherapy, insight oriented, behavior modifying and/or supportive, in an inpatient hospital, partial hospital or residential care setting, approximately 75 to 80 minutes face-to-face with the patient; with medical evaluation and management services |
| 90823 | Individual psychotherapy, interactive, using play equipment, physical devices, language interpreter, or other mechanisms of non-verbal communication, in an inpatient hospital, partial hospital or residential care setting, approximately 20 to 30 minutes face-to-face with the patient |
| 90824 | Individual psychotherapy, interactive, using play equipment, physical devices, language interpreter, or other mechanisms of non-verbal communication, in an inpatient hospital, partial hospital or residential care setting, approximately 20 to 30 minutes face-to-face with the patient; with medical evaluation and management services |
| 90826 | Individual psychotherapy, interactive, using play equipment, physical devices, language interpreter, or other mechanisms of non-verbal communication, in an inpatient hospital, partial hospital or residential care setting, approximately 45 to 50 minutes face-to-face with the patient |
| 90827 | Individual psychotherapy, interactive, using play equipment, physical devices, language interpreter, or other mechanisms of non-verbal communication, in an inpatient hospital, partial hospital or residential care setting, approximately 45 to 50 minutes face-to-face with the patient; with medical evaluation and management services |
| 90828 | Individual psychotherapy, interactive, using play equipment, physical devices, language interpreter, or other mechanisms of non-verbal communication, in an inpatient hospital, partial hospital or residential care setting, approximately 75 to 80 minutes face-to-face with the patient |
| 90829 | Individual psychotherapy, interactive, using play equipment, physical devices, language interpreter, or other mechanisms of non-verbal communication, in an inpatient hospital, partial hospital or residential care setting, approximately 75 to 80 minutes face-to-face with the patient; with medical evaluation and management services |
| 90832 | Psychotherapy, 30 minutes with patient and/or family member |
| 90833 | Psychotherapy, 30 minutes with patient and/or family member when performed with an evaluation and management service (List separately in addition to the code for primary procedure) |
| 90834 | Psychotherapy, 45 minutes with patient and/or family member |
| 90836 | Psychotherapy, 45 minutes with patient and/or family member |
| 90837 | Psychotherapy, 60 minutes with patient and/or family member |
| 90838 | Psychotherapy, 60 minutes with patient and/or family member when performed with an evaluation and management service (List separately in addition to the code for primary procedure) |
| 90839 | Psychotherapy for crisis; first 60 minutes |
| 90840 | Psychotherapy for crisis; each additional 30 minutes (List separately in addition to code for primary service) |
| 90845 | Psychoanalysis |
| 90846 | Family psychotherapy (without the patient present) |
| 90847 | Family psychotherapy (conjoint psychotherapy) (with patient present) |
| 90849 | Multiple-family group psychotherapy |
| 90853 | Group psychotherapy (other than of a multiple-family group) |
| 90857 | Interactive group psychotherapy |
| 96152 | Health and behavior intervention, each 15 minutes, face-to-face; individual |
| 96153 | Health and behavior intervention, each 15 minutes, face-to-face; group (2 or more patients) |
| 96154 | Health and behavior intervention, each 15 minutes, face-to-face; family (with the patient present) |
| 96155 | Health and behavior intervention, each 15 minutes, face-to-face; family (without the patient present) |
| 97532 | Development of cognitive skills to improve attention, memory, problem solving (includes compensatory training), direct (one-on-one) patient contact, each 15 minutes |
| 97533 | Sensory integrative techniques to enhance sensory processing and promote adaptive responses to environmental demands, direct (one-on-one) patient contact, each 15 minutes |
| 99510 | Home visit for individual, family, or marriage counseling |
| G0409 | Social work and psychological services, directly relating to and/or furthering the patient's rehabilitation goals, each 15 minutes, face-to-face; individual (services provided by a CORF qualified social worker or psychologist in a CORF) |
| G0410 | Group psychotherapy other than of a multiple-family group, in a partial hospitalization setting, approximately 45 to 50 minutes |
| G0411 | Interactive group psychotherapy, in a partial hospitalization setting, approximately 45 to 50 minutes |
| H0004 | Behavioral health counseling and therapy, per 15 minutes |
| H0035 | Mental health partial hospitalization, treatment, less than 24 hours |
| H0036 | Community psychiatric supportive treatment, face-to-face, per 15 minutes |
| H2012 | Behavioral health day treatment, per hour |
| H2014 | Skills training and development, per 15 minutes |
| H2015 | Comprehensive community support services, per 15 minutes |
| H2017 | Psychosocial rehabilitation services, per 15 minutes |
| H2019 | Therapeutic behavioral services, per 15 minutes |
| H2021 | Community-based wrap-around services, per 15 minutes |
| H2027 | Psychoeducational service, per 15 minutes |
| H2030 | Mental health clubhouse services, per 15 minutes |
| H2032 | Activity therapy, per 15 minutes |
| H2033 | Multisystemic therapy for juveniles, per 15 minutes |
| T1027 | Family training and counseling for child development, per 15 minutes |
| Z1833 | Counseling and therapy, individual |
| Z1834 | Counseling and therapy, group |
| Z1840 | Community Psychiatric Support treatment, individual |
| Z1841 | Community Psychiatric Support treatment, group |

### Table A3. Census Tract Urbanicity Classification

| Rural-Urban Continuum Code | Description | Classification |
| --- | --- | --- |
| 1 | Counties in metro areas of 1 million population or more | Urban |
| 2 | Counties in metro areas of 250,000 to 1 million population | Urban |
| 3 | Counties in metro areas of fewer than 250,000 population | Urban |
| 4 | Urban population of 20,000 or more, adjacent to a metro area | Urban |
| 5 | Urban population of 20,000 or more, not adjacent to a metro area | Urban |
| 6 | Urban population of 2,500 to 19,999, adjacent to a metro area | Rural |
| 7 | Urban population of 2,500 to 19,999, not adjacent to a metro area | Rural |
| 8 | Completely rural or less than 2,500 urban population, adjacent to a metro area | Rural |
| 9 | Completely rural or less than 2,500 urban population, not adjacent to a metro area | Rural |

### Table A4. Taxonomy Code Categorization

|  | Subcategory | Entity | Included Provider Types or Classifications | Excluded Classifications or Specializations |
| --- | --- | --- | --- | --- |
| Mental Health | Psychiatrist (PST) | 1 | Psychiatry & Neurology | Clinical Neurophysiology, Vascular Neurology, Diagnostic Neuroimaging, Sports Medicine, Pain Medicine, Neurodevelopmental Disabilities |
|  | Psychologist (PSG) | 1 | Psychologists, Clinical Neuropsychologists |  |
|  | Counselor (CLR) | 1 | Counselors |  |
|  | Social Worker (SW) | 1 | Social Workers |  |
|  | Other Entity 1 Mental Health (OM1) | 1 | Marriage and Family Therapists, Psychoanalysts, Behavior Analysts, Psychiatric/Mental Health Registered Nurses, Psychiatric/Mental Health Nurse Practitioners, Psychiatric/Mental Health Clinical Nurse Specialists |  |
|  | Entity 2 Mental Health (MHE2) | 2 | Categories above combined | Categories above combined |
|  | Mental Health Center (MHC) | 1 or 2 | Community/Behavioral Health Agencies; Mental Health Clinic/Centers; Psychiatric, Mental Illness, and Emotionally Disturbed Children Residential Treatment Facilities; Psychiatric Hospitals; Psychiatric Units |  |

## References

1. PD&R. *HUD USPS ZIP Code Crosswalk Files*. 2018 [cited 2023; Available from: <https://www.huduser.gov/portal/datasets/usps_crosswalk.html>.

2. Agriculture, U.S.D.o. *USDA ERS - Rural-Urban Commuting Area Codes*. 2018 2023]; Available from: <https://www.ers.usda.gov/data-products/rural-urban-commuting-area-codes/>.

3. NUCC. *Health Care Provider Taxonomy*. 2018 [cited 2023; Available from: <https://www.nucc.org/index.php/code-sets-mainmenu-41/provider-taxonomy-mainmenu-40>.
