# Supplementary material for: Evaluating access to psychosocial services for the medicaid-insured children in Georgia
Source: BMC Public Health. 2025 Jan 20;25:244. doi: 10.1186/s12889-025-21374-7 (PMC11748264; doi:10.1186/s12889-025-21374-7)
Supplement: Supplementary file 2 — Supplementary Material 2 [file 12889_2025_21374_MOESM2_ESM.docx]

Online-Supplement B

This online supplement includes details about the access model, outcome measures, and intervention analysis.

Table of Contents

[Access Model 2](#_Toc145951437)

[Objective 2](#_Toc145951438)

[Constraints 2](#_Toc145951439)

[Variables/Auxiliary Variables 3](#_Toc145951440)

[Inputs and Parameters 3](#_Toc145951441)

[Outputs 5](#_Toc145951442)

[Outcome Measures 6](#_Toc145951443)

[Figure B1. Flowchart of One-way Travel Distance Computation 7](#_Toc145951444)

[Figure B2. Flowchart of Tract-level Percent-met Demand Computation 7](#_Toc145951445)

[Figure B3. Flowchart of Tract-level Service Coverage Computation 7](#_Toc145951446)

[Intervention Analysis 8](#_Toc145951447)

[Figure B4. Flowchart of Caseload Intervention (Random Approach) 9](#_Toc145951448)

[Figure B5. Flowchart of Caseload Intervention (Targeted Approach) 10](#_Toc145951449)

[Figure B6. Flowchart of Workforce Intervention (Random Approach) 11](#_Toc145951450)

[Figure B7. Flowchart of Workforce Intervention (Targeted Approach) 12](#_Toc145951451)

[Figure B8. Flowchart of In-home Intervention 13](#_Toc145951452)

[References 14](#_Toc145951453)

## Access Model

We modeled MH access using an optimization approach, which assigns caseloads provided by MH providers to serve the estimated demand from patients in each census tract.

### Objective

The objective of this model aimed to minimize the total travel distance from in-clinic assignments. The travel distance revealed the accessibility of patients getting in-clinic MH services. Additionally, this objective value also penalized for large differences in average travel distances between neighboring census tracts. Together, we wanted to find the best, balanced assignments for in-clinic and in-home caseloads such that patients travel could travel the least distance to receive care.

$${min}_{y,z} \left( 1-\lambda\right)\left[ \sum_{i\in S} \sum_{j\in P} d_{ij}y_{ij} \right]+ \lambda\left[ \sum_{i_{1},i_{2}\in S: d_{i_{1},i_{2}}^{C}\leq d_{pen}^{Max}} \frac{\left( w_{i_{1}}-w_{i_{2}} \right)^{2}}{d_{i_{1},i_{2}}^{C}} \right]$$

### Constraints

The following constraints collectively aimed to replicate the scenarios of patients accessing MH services in real-life.

| Constraint |  | # | Description |
| --- | --- | --- | --- |
| $w_{i}=\frac{1}{V_{i}}\left[ \sum_{j\in P} d_{ij}y_{ij}+d^{Max}\times\left( V_{i}-\sum_{j\in P} x_{ij} \right) \right]$ | $\forall i\in S$ | 1 | Set the average travel distance used in the objective. Average travel distance is calculated by averaging the total travel distance plus the total unassigned visit distance |
| $\sum_{j\in P} x_{ij}\leq V_{i}$ | $\forall i\in S$ | 2 | For each census tract $i$, the number of total visits assigned is less than the total needs |
| $x_{ij}=y_{ij}+z_{ij}+u_{ij}$ | $\forall i\in S$  $\forall j\in P$ | 3 | Total number of visits between census tract $i$ and provider $j$ is the summation of in-clinic, in-home, and in-school visits between census tract $i$ and provider $j$ |
| $\sum_{i\in S} \sum_{j\in P} x_{ij}\geq c^{min}\times\sum_{i\in S} V_{i}$ |  | 4 | At least a given percentage of the total needs is covered with our assignment |
| $\sum_{j\in P:d_{ij}>d^{Max}} y_{ij}=0$ | $\forall i\in S$ | 5 | For each census tract $i$, assume no in-clinic visits if the provider location $j$ is farther than $d^{Max}$ |
| $\sum_{i\in S} y_{ij}\leq{cap}_{j}\times q^{O}$ | $\forall j\in P$ | 6 | For each in-clinic provider location $j$, assume the total number of in-clinic visits to be less than the observed total visits scaled up by a no-show factor $q^{O}$ |
| $\sum_{i\in C_{j}} z_{ij}\leq h_{j}$ | $\forall j\in P$ | 7 | The total number of assigned in-home visits from counties served by provider $j$ should be less than the total capacity of provider $j$ |
| $y_{ij},z_{ij}\geq0$ | $\forall i\in S$  $\forall j\in P$ | 8 | Non negativity assumption for our decision variable |

### Variables/Auxiliary Variables

| Variable | Description |
| --- | --- |
| $x_{ij}$ | Number of visits between census tract $i$ and provider $j$ (auxiliary) |
| $y_{ij}$ | Number of in-clinic visits between census tract $i$ and provider $j$ |
| $z_{ij}$ | Number of in-home visits between census tract $i$ and provider $j$ |
| $w_{i}$ | Average travel distance for patients in census tract $i$ (auxiliary) |

### Inputs and Parameters

| Parameter | Description | Value | Source/Explanation |
| --- | --- | --- | --- |
| $\lambda$ | Trade-off parameter, defines the relative importance | 0.55 |  |
| $d_{i_{1},i_{2}}^{C}$ | Travel distance between centroids of census tract $i_{1}$ and $i_{2}$ |  | Census tract centroids obtained from Census Data 2017.  Travel distance calculated using ArcGIS |
| $d_{pen}^{Max}$ | Max distance (miles) between two census tract centroids to be considered neighboring | 10 |  |
| $V_{i}$ | Demand from census tract $i$ |  | MH demand (See Figure A1 in Online-Appendix A for procedures calculating MH demand) |
| $d_{ij}$ | Travel distance between census tract $i$ (centroid) and provider $j$ |  | Provider practice location addresses from MH supply (See Figure A2 in Online-Appendix A for procedures obtaining MH provider practice location addresses.  Travel distance calculated using ArcGIS |
| $d^{Max}$ | Max allowed distance between a patient and a matched provider | 65 |  |
| $u_{ij}$ | Number of observed in-school visits between census tract $i$ and provider $j$ |  | In-school assignments (See Figure A5 in Online-Appendix A for procedures obtaining MH in-school assignments) |
| $c^{min}$ | Minimum percentage of the total patient population required to be assigned to providers.  The model becomes infeasible if this parameter is set too high. |  | $\frac{\sum_{j\in P} {(cap}_{j}+h_{j})}{\sum_{i\in S} {(V}_{i}-\sum_{j\in P} u_{ij})}$  Start at the value above, which is the total number of MH caseloads available to cover MH demands. |
| ${cap}_{j}$ | Max number of yearly in-clinic visits of provider$j$ |  | In-clinic MH caseloads (See Figure A2 in Online-Appendix A for procedures obtaining MH in-clinic caseloads) |
| $q^{O}$ | No-show scaling factor for in-clinic visits | 1.1 | 10% no-show, representing 10% of the child patients failed to show up for their in-clinic appointments |
| $h_{j}$ | Max number of yearly in-home visits for provider $j$ |  | In-home MH caseloads (See Figure A2 in Online-Appendix A for procedures obtaining MH in-home caseloads) |

|  | Description | Source/Explanation |
| --- | --- | --- |
| $S$ | The set of census tracts in Georgia | From Census data 2017 |
| $P$ | The set of provider locations in Georgia | From in-clinic and in-home supply (See Figure A2 in Online-Appendix A for procedures obtaining MH provider practice location addresses) |
| $C_{j}$ | The set of counties covered by in-home provider $j$ | Each census tract can only be in one county. Equivalently, since we want $i\in C_{j}$, we can obtain the list of census tracts covered by provider $j$ instead. See Figure A4 for procedures to retrieve the list of census tracts covered by in-home provider practices |

### Outputs

The outputs include the values for $y_{ij}$ and $z_{ij}$, which we referred to as the caseload assignment from provider $j$ to serve the demand in census tract $i$. According to the recommendations for public health access from the Department of Health and Human Services [1], patients should not travel more than 45 miles if they live in a rural census tract and 30 miles if in an urban census tract. We further remove the related assignments if the travel distances exceed this recommendation.

Figure B1. Flowchart of One-way Travel Distance Computation


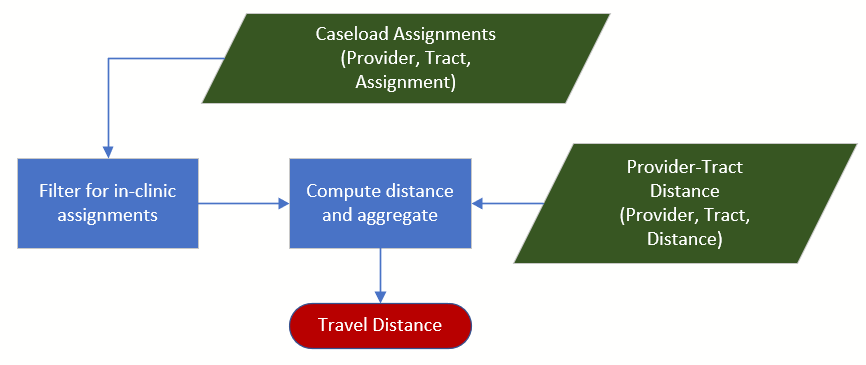


Note: In travel distance computation we only considered in-clinic assignments. We first computed the distance traveled from each in-clinic assignment, then we aggregated the total travel distance for all assignments.

### Figure B2. Flowchart of Tract-level Percent-met Demand Computation


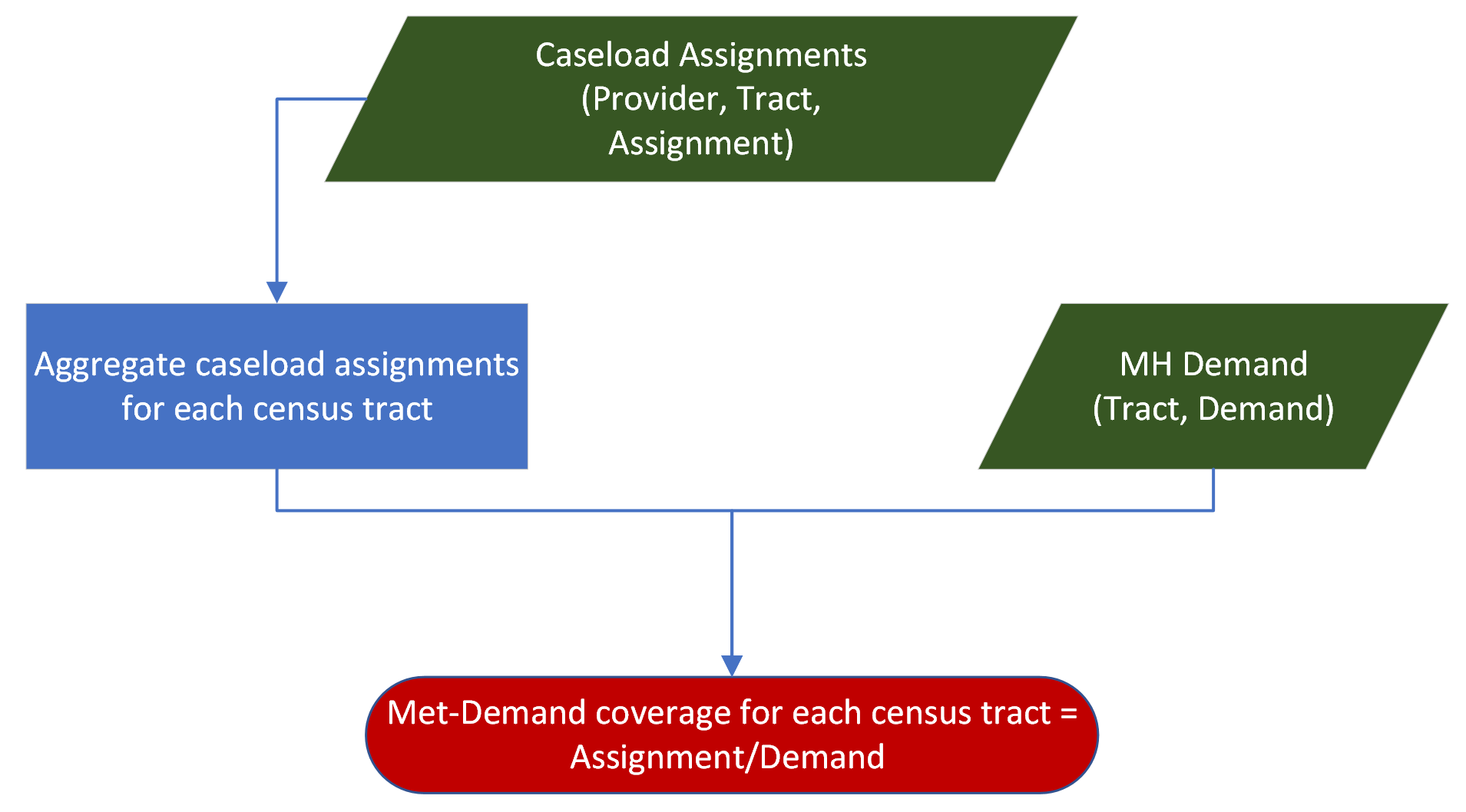


Note: We aggregated the total assignment from each census tract, then we computed its percentage over the estimated MH demand from corresponding census tract.

### Figure B3. Flowchart of Tract-level Service Coverage Computation


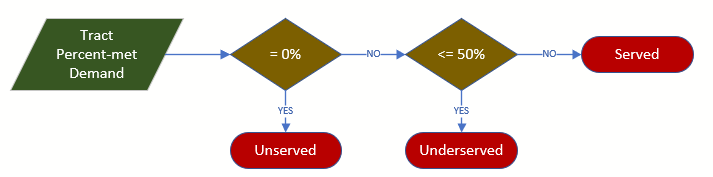


Note: We considered census tracts with 0% coverage as unserved tracts; with 50% or less coverage as underserved tracts; and the remainder as served tracts.

## Intervention Analysis

Together, we proposed three interventions to lower the barriers for MH patients accessing adequate psychosocial services:

1. Caseload intervention: illustrated in Figure B4 and Figure B5, caseload intervention targeted supply shortage and aimed to increase the availability of caseloads from existing MH practices.
2. Workforce intervention: illustrated in Figure B6 and Figure B7, workforce intervention targeted supply shortage and aimed to increase caseload availability by bringing non-Medicaid-participating MH practices into Medicaid.
3. In-home intervention: illustrated in Figure B8, in-home intervention targeted lowering the travel distance by promoting in-clinic practices to provide in-home cares.

We varied the level of increase ($X\%$) of total caseloads for caseload and workforce intervention and sampled MH practices to elevate supply with the following two approaches:

1. Random approach: illustrated in Figure B4 and Figure B6, randomly sample MH providers to increase caseload.
2. Targeted approach: illustrated in Figure B5 and Figure B7, sample MH providers near unserved or underserved census tracts to increase caseload.

### Figure B4. Flowchart of Caseload Intervention (Random Approach)


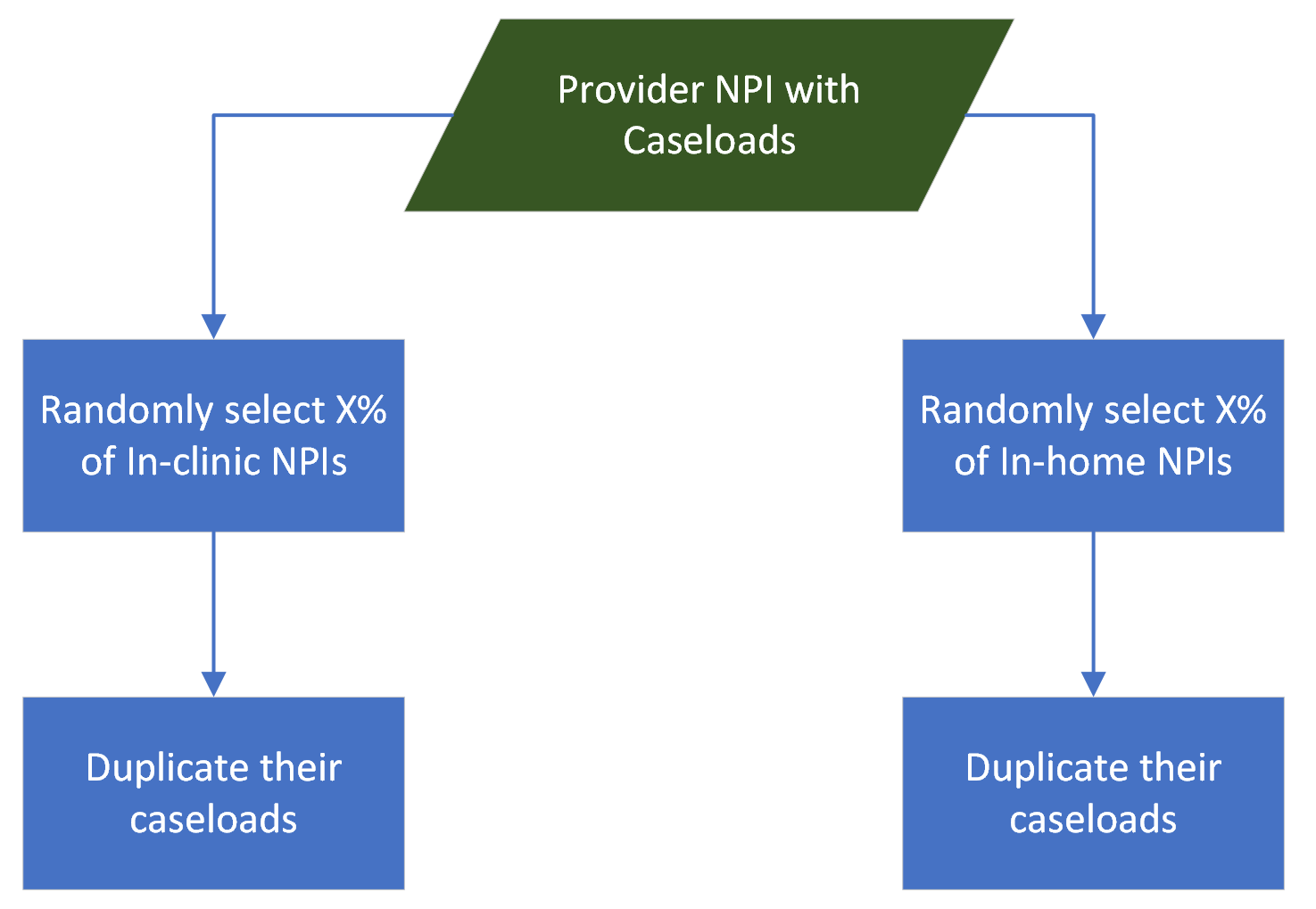


### Figure B5. Flowchart of Caseload Intervention (Targeted Approach)


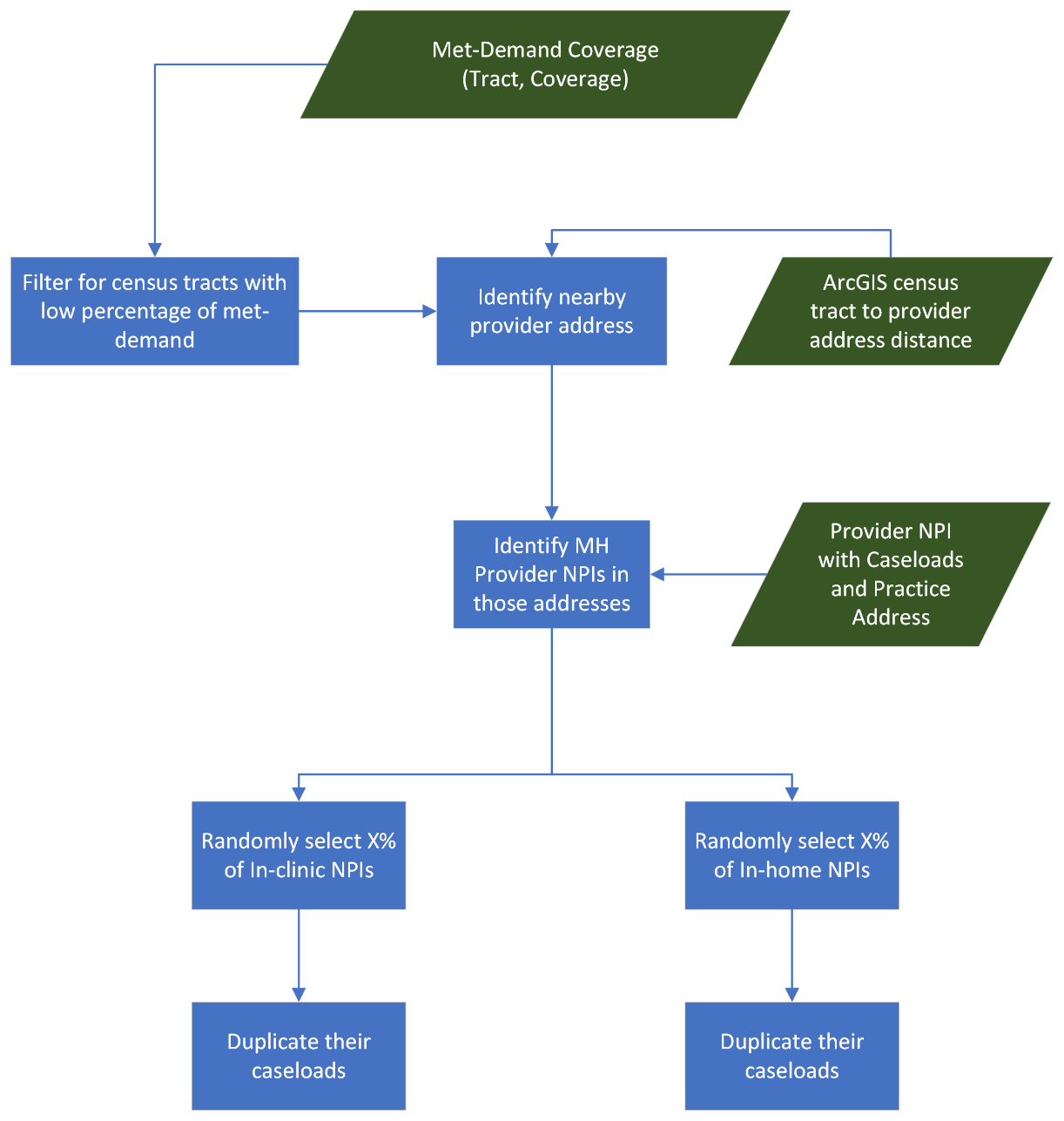


### Figure B6. Flowchart of Workforce Intervention (Random Approach)


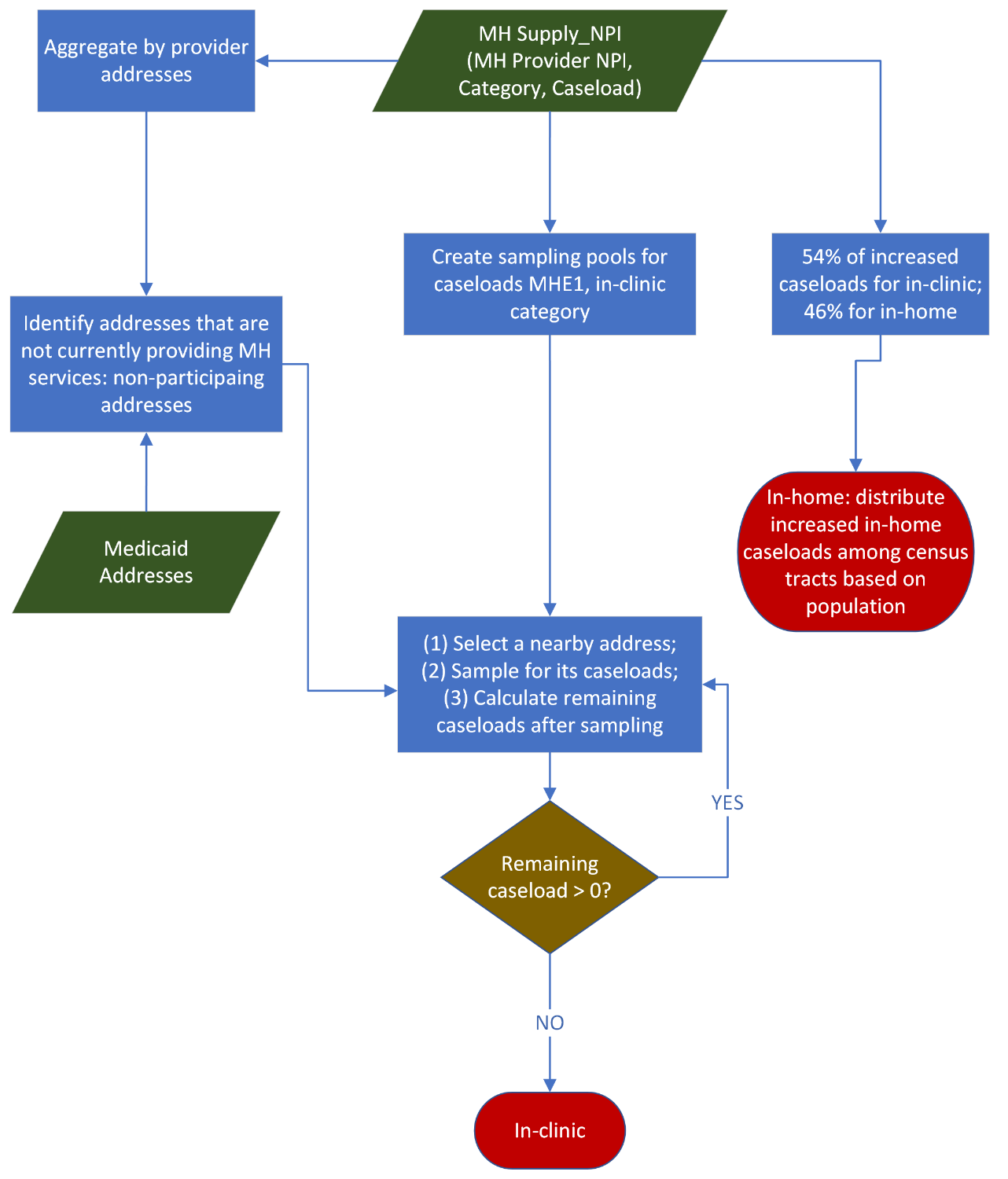


### Figure B7. Flowchart of Workforce Intervention (Targeted Approach)


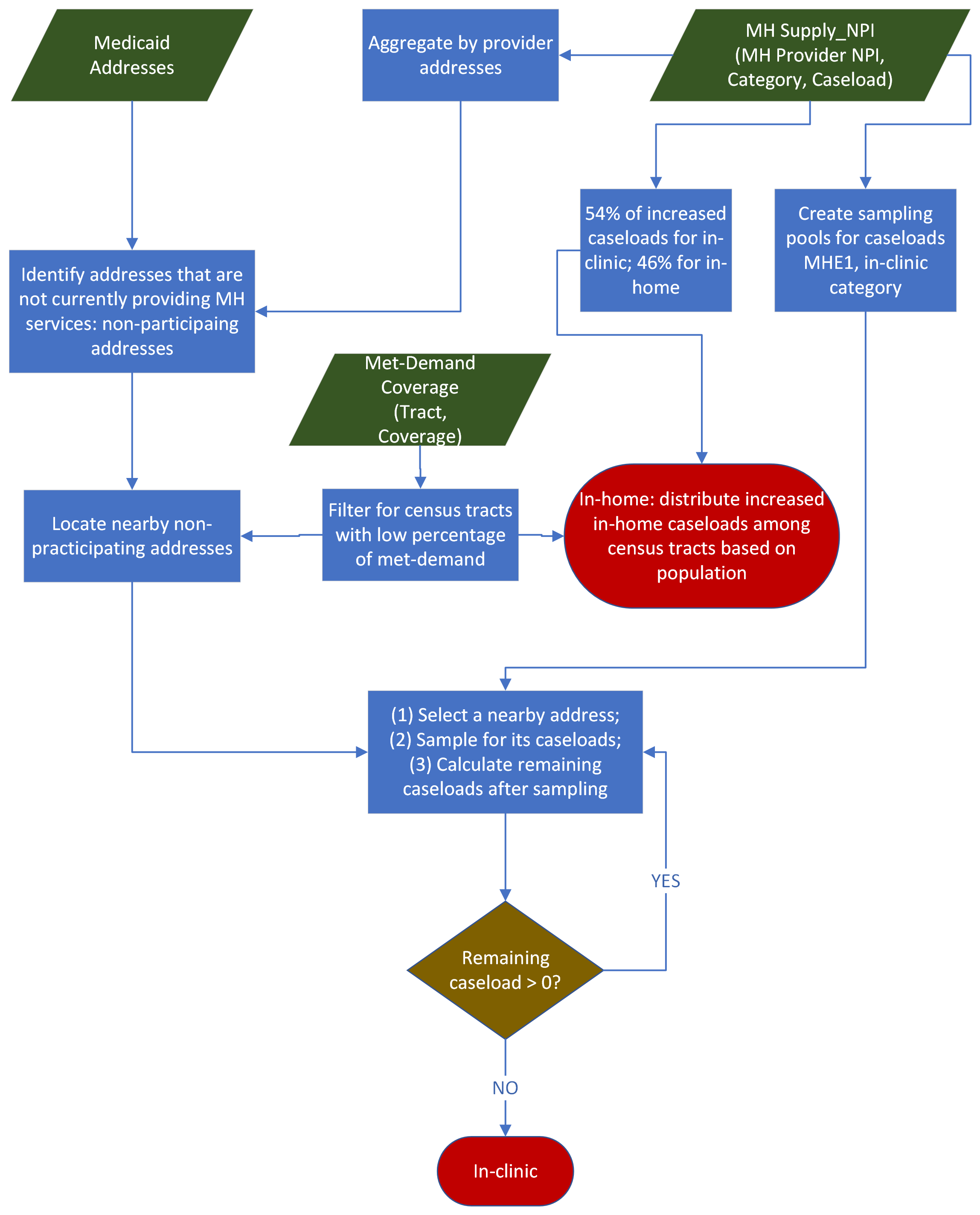


### Figure B8. Flowchart of In-home Intervention


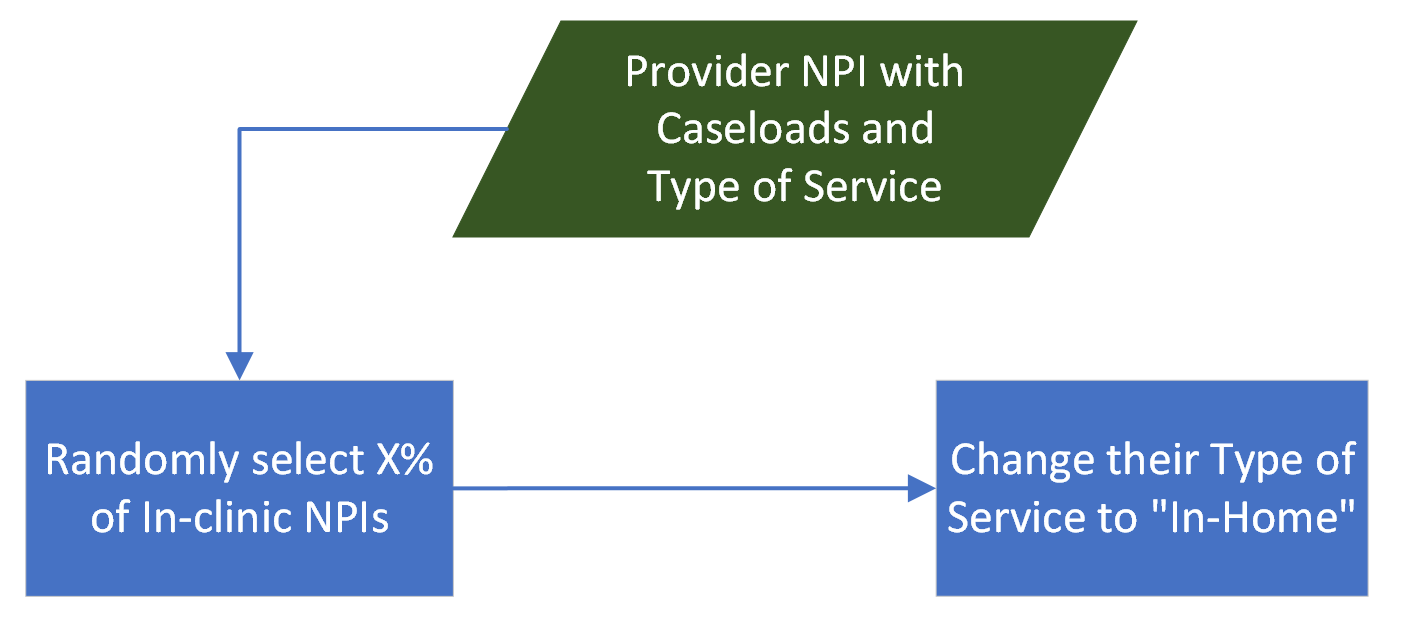


## References

1. Murrin, S., *State Standards for Access to Care in Medicaid Managed Care*. 2014, Department of Health and Human Services. p. 22.
