# Supplementary material for: Evaluating access to psychosocial services for the medicaid-insured children in Georgia
Source: BMC Public Health. 2025 Jan 20;25:244. doi: 10.1186/s12889-025-21374-7 (PMC11748264; doi:10.1186/s12889-025-21374-7)
Supplement: Supplementary file 3 — Supplementary Material 3 [file 12889_2025_21374_MOESM3_ESM.docx]

Online-Supplement C

This online supplement includes details about the analysis on study population, demand, supply, and outcome measures from access modeling and intervention. The following supplemental material is included in this supplement:

Table of Contents

[Analysis on Demographics 2](#_Toc155708462)

[Figure C1. Study Population Demographics (Sex, Race/Ethnicity) 3](#_Toc155708463)

[Figure C2. Study Population Demographics (Age) 4](#_Toc155708464)

[Figure C3. Demand Demographic 5](#_Toc155708465)

[Figure C4. Enrollment Demographic 6](#_Toc155708466)

[Analysis on Demand 7](#_Toc155708467)

[Figure C5. Estimated Demand 7](#_Toc155708468)

[Analysis on Supply 8](#_Toc155708469)

[Figure C6. Supply Analysis 8](#_Toc155708470)

[Analysis on Access 9](#_Toc155708471)

[Tables 10](#_Toc155708472)

[Table C1. Demand Demographics 10](#_Toc155708473)

[Table C2. Estimated Demand 10](#_Toc155708474)

[Table C3. Supply 11](#_Toc155708475)

[Table C4. Access Results (Difference from Baseline) 12](#_Toc155708476)

## Analysis on Demographics

We performed various types of analysis on the demographics of our study population.

In Figure C1 and C2, we analyzed the breakdowns our study population from three aspects:

1. Sex: male or female
2. Race/ethnicity: (1) AIAN: American Indian and Alaska Natives; (2) Asian; (3) Black; (4) Hawaiian/Pacific Islander; (5) Hispanic; (6) White
3. Age group: (1) pre-school: 3-4; (2) children: 5-12; and (3) adolescent: 13-18

In Figure C3, we analyzed the breakdowns of average demand (i.e., demand over study population) by the same three aspects, particularly including the trends of %study population as age grows.

In Figure C4, we looked at the enrollment statues from the same three aspects.

Numerical details were displayed in Tables C1.

### Figure C1. Study Population Demographics (Sex, Race/Ethnicity)

Among all Medicaid-insured childre, 21% were included as our study population. This ratio was higher among male (23%) compared to female (20%); it was highest among White race/ethnicity group while lowest among Hispanic group.

Within our study population, we observed an even split (50.91% male vs. 49.09% female) between the two different sex. The majority of the study population belonged to White (46.07%) and Black (48.77%) race/ethnicity groups.


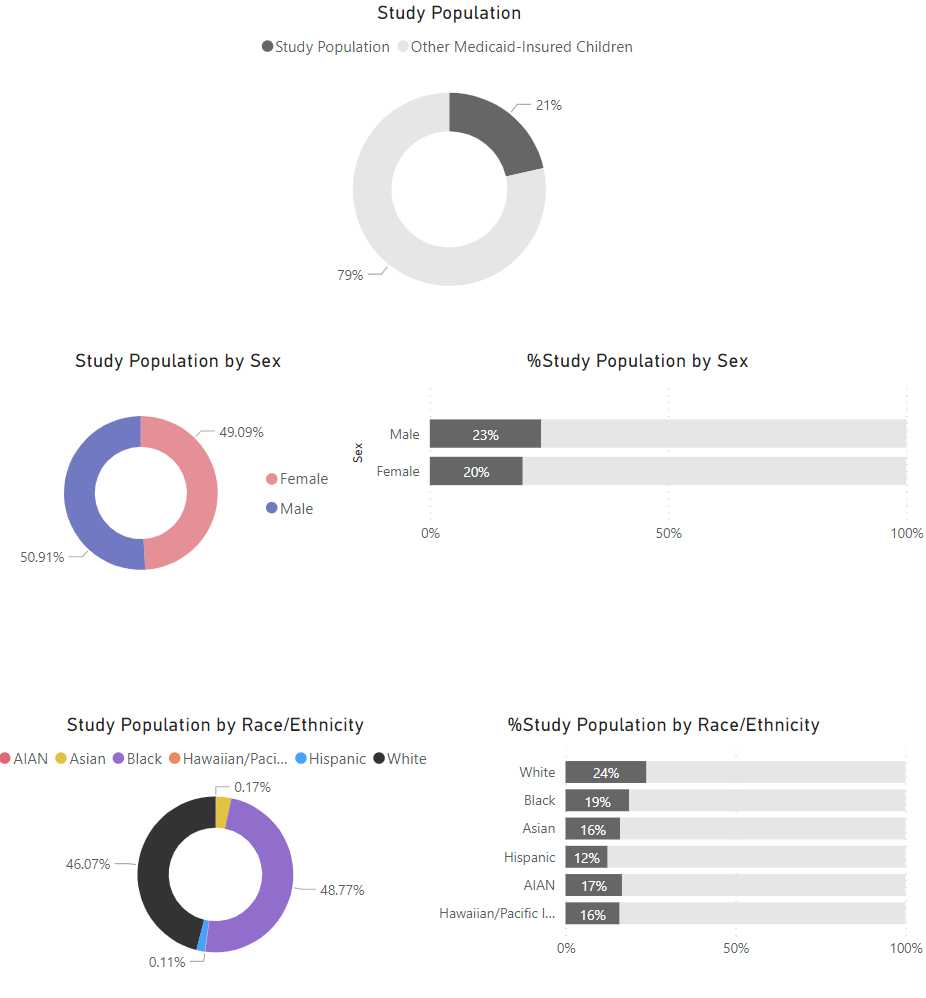


### Figure C2. Study Population Demographics (Age)

The percentage of study population (over all Medicaid-insured children) increased as age increases: from 13% in pre-school to 26% in adolescent. Further breaking down by sex or race/ethnicity preserved this trend.


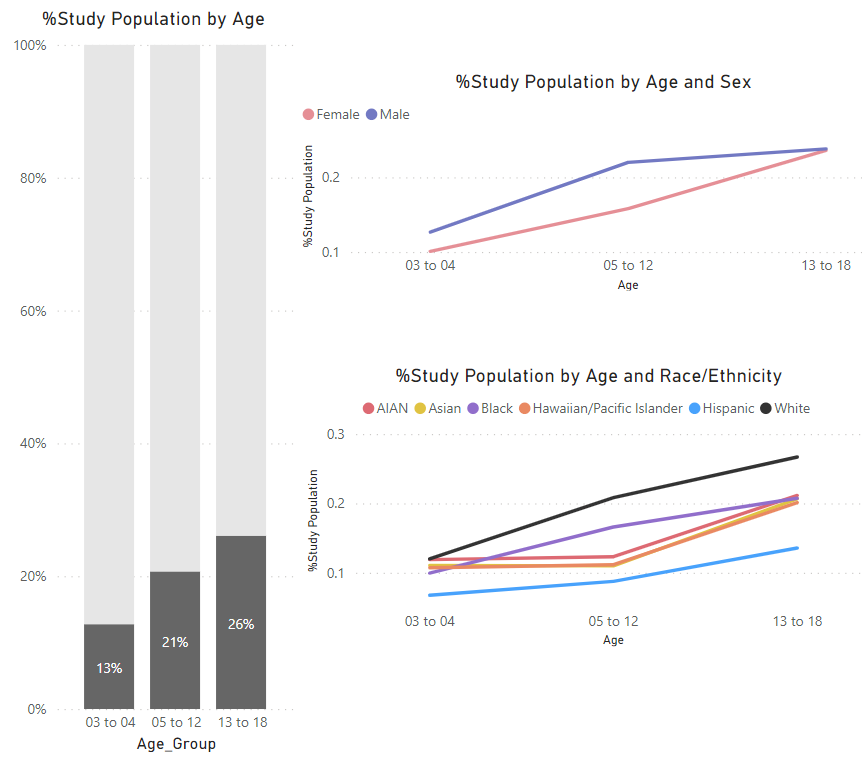


### Figure C3. Demand Demographic

The average demand differed among different sex, age, and race/ethnicity groups. We also observed a “jump” in average demand from pre-school age to children age.


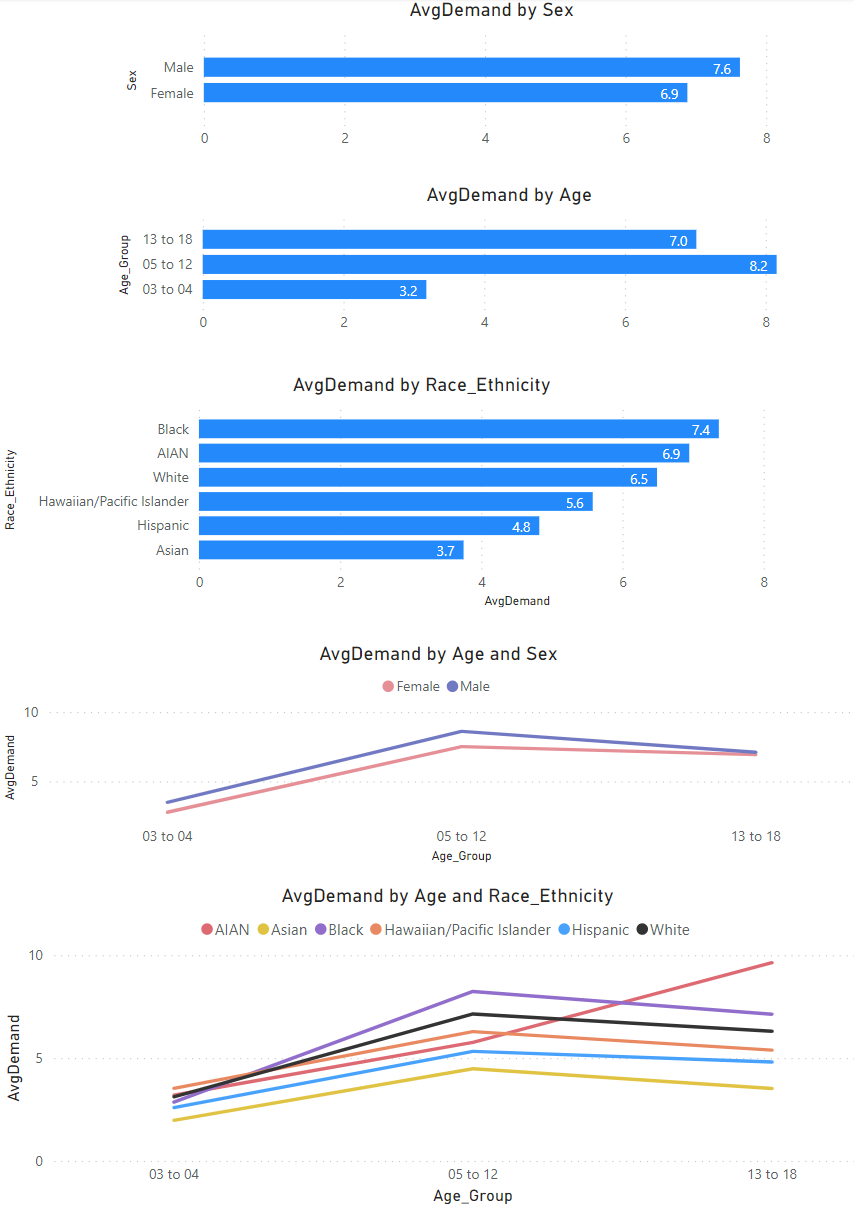


### Figure C4. Enrollment Demographic

Our population was corrected using Medicaid enrollment. We observed the enrollment adjustment was similar among sex and age while Hispanic race/ethnicity group showed lower enrollment among all groups.


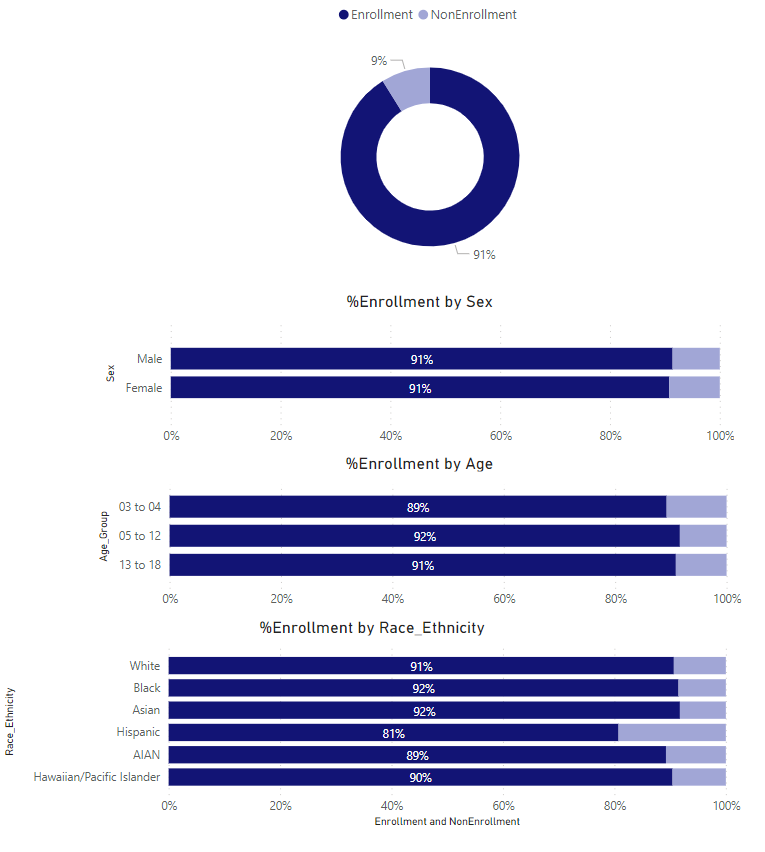


## Analysis on Demand

We analyzed our MH prevalence and estimated demand by urbanicity in Figure C5.

Numerical details were presented in Table C2.

### Figure C5. Estimated Demand

We observed that majority of the demand and MH-diagnosed children came from urban census tracts.


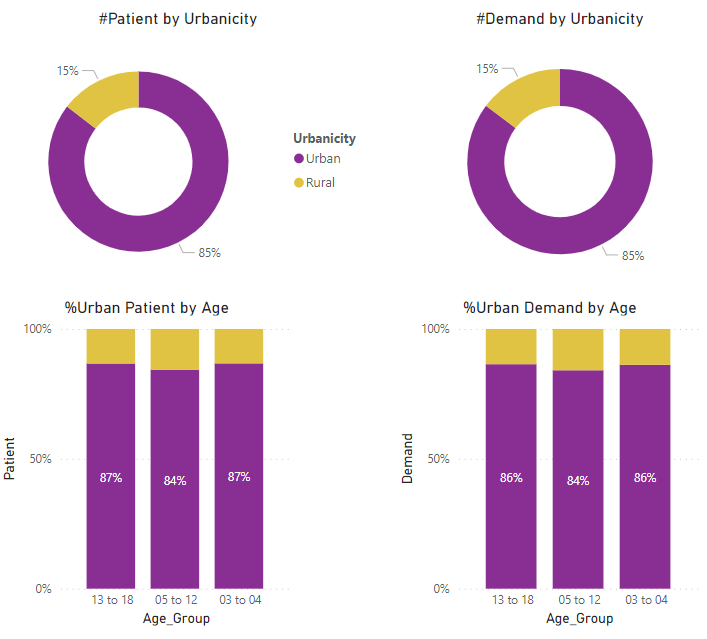


## Analysis on Supply

We analyzed the provider in Figure C6 for:

1. Medicaid participation
2. Provider classifications: Entity type 1 (individual practitioner) vs. type 2 (organization); MHE1 vs. MHE2.
3. Caseload classifications: delivered under in-clinic, in-home, or in-school care settings.

Numerical details were presented in Table C3.

### Figure C6. Supply Analysis

We analyzed all available NPPES in 2017, and observed that only 13% of them had provided psychosocial services under Medicaid in 2018. Majority (83%) of these providers were entity 1 and were classified as MHE1. They had provided caseloads mostly (66%) under in-clinic settings, with some (24%) in-home deliveries, and few (1%) in school.


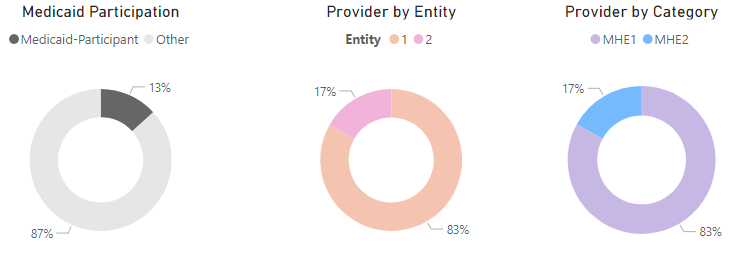


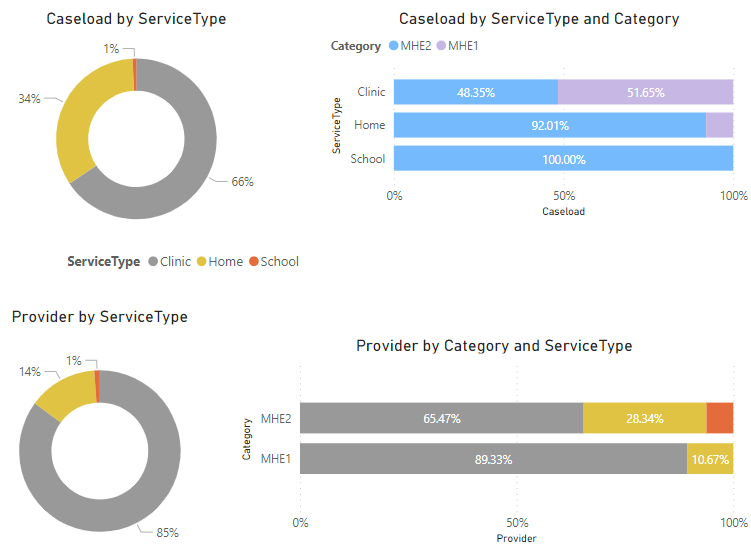


## Analysis on Access

Table C4 displayed the numerical details of access model outcome measures, including:

1. Travel distance: one-way travel (in miles) to receive psychosocial services.
2. Percent-met demand: the percentage of demand met by the available caseload from MH practices.
3. Service coverage: categorization of census tracts into (1) unserved tracts with 0% met demand; (2) underserved tracts with 50% or less percent-met demand (but not zero); and (3) served tracts, the remainder.

Alongside the baseline outcome measures, we presented the outcomes measures for our different interventions, including:

1. Caseload intervention
2. Workforce intervention
3. In-home intervention

Under random and/or targeted approach(es).

The numbers for all interventions were presented as their difference compared to baseline numbers.

## Tables

### Table C1. Demand Demographics

| Sex | Race Ethnicity | Age 3-4 | | | | Age 5-12 | | | | Age 13-18 | | | |
| --- | --- | --- | --- | --- | --- | --- | --- | --- | --- | --- | --- | --- | --- |
|  |  | Medicaid-Insured | Study Population | MH Patient | Avg Demand | Medicaid-Insured | Study Population | MH Patient | Avg Demand | Medicaid-Insured | Study Population | MH Patient | Avg Demand |
| Female | AIAN | 187 | 20 | 17 | 2.30 | 528 | 58 | 52 | 5.09 | 308 | 71 | 65 | 9.63 |
|  | Asian | 2030 | 244 | 219 | 1.78 | 9222 | 1005 | 919 | 4.37 | 7289 | 1699 | 1567 | 3.48 |
|  | Black | 38899 | 3934 | 3529 | 2.53 | 157635 | 23642 | 21739 | 7.70 | 102288 | 23052 | 20991 | 6.87 |
|  | H/P | 78 | <11 | <11 | 1.50 | 319 | 31 | 26 | 5.13 | 239 | 56 | 53 | 5.50 |
|  | Hispanic | 1472 | 115 | 82 | 1.61 | 5444 | 492 | 385 | 4.54 | 3400 | 530 | 435 | 3.92 |
|  | White | 37174 | 4594 | 4079 | 2.88 | 146425 | 29017 | 26399 | 6.79 | 92677 | 27918 | 25188 | 6.58 |
|  | Unknown | 7254 | 965 | 852 | 3.62 | 26764 | 5736 | 5250 | 11.18 | 16878 | 4852 | 4398 | 10.74 |
| Male | AIAN | 208 | 32 | 30 | 3.75 | 503 | 88 | 75 | 6.20 | 152525 | 39392 | 35904 | 7.40 |
|  | Asian | 2135 | 281 | 242 | 2.14 | 10108 | 1305 | 1206 | 4.57 | 283 | 66 | 60 | 9.64 |
|  | Black | 38682 | 4651 | 4191 | 3.14 | 155858 | 32929 | 30323 | 8.63 | 7612 | 1639 | 1511 | 3.57 |
|  | H/P | 90 | 13 | 13 | 4.46 | 344 | 53 | 48 | 6.96 | 101245 | 23254 | 21217 | 7.40 |
|  | Hispanic | 1558 | 160 | 123 | 3.29 | 5918 | 756 | 610 | 5.84 | 243 | 50 | 44 | 5.26 |
|  | White | 38667 | 5641 | 5019 | 3.30 | 152525 | 39392 | 35904 | 7.40 | 94236 | 27217 | 24703 | 6.02 |
|  | Unknown | 9285 | 2035 | 1849 | 5.02 | 36555 | 12182 | 11450 | 13.06 | 21446 | 6856 | 6384 | 11.30 |

### Table C2. Estimated Demand

| Urbanicity | Age 3-4 | | Age 5-12 | | Age 13-18 | |
| --- | --- | --- | --- | --- | --- | --- |
|  | MH Patient | Estimated Demand | MH Patient | Estimated Demand | MH Patient | Estimated Demand |
| Urban | 20333 | 121162 | 123504 | 955073 | 101858 | 906582 |
| Rural | 3104 | 19384 | 22991 | 180551 | 15628 | 142268 |

### Table C3. Supply

| Category | Entity | Service Type | Potential Provider | Potential Practice | Actual Provider | Actual Practice | Patient | Caseload |
| --- | --- | --- | --- | --- | --- | --- | --- | --- |
| MHE1 | 1 | Clinic | 15927 | 7109 | 2051 | 1429 | 99312 | 495643 |
|  |  | Home |  |  | 245 | 232 | 3719 | 39259 |
| MHE2 | 1 | Clinic | 161 | 80 | 7 | 7 | 98 | 782 |
|  |  | Home |  |  | 1 | 1 | 18 | 123 |
|  | 2 | Clinic | 4767 | 3536 | 321 | 304 | 53488 | 463191 |
|  |  | Home |  |  | 141 | 139 | 28546 | 451845 |
|  |  | School | N/A | N/A | 31 | 130 | 11082 | 11082 |

### Table C4. Access Results (Difference from Baseline)

| Intervention | Sampling Approach | Sampling Ratio | Urban Travel Distance per Visit (miles) | Rural Travel Distance per Visit (miles) | Urban Percent-Met Demand (%) | Rural Percent-Met Demand (%) | Urban Unserved Tract (%) | Rural Unserved Tract (%) | Urban Underserved Tract (%) | Rural Underserved Tract (%) |
| --- | --- | --- | --- | --- | --- | --- | --- | --- | --- | --- |
| **No Intervention (Baseline)** | | | **3.60** | **6.84** | **72.72** | **27.07** | **4.64** | **47.35** | **10.72** | **31.79** |
| Caseload | Random | 5 | 0.13 | 1.83 | 3.77 | 5.33 | -0.66 | -6.29 | -1.87 | 1.32 |
|  |  | 10 | 0.19 | 1.67 | 6.89 | 7.64 | -0.96 | -7.62 | -3.25 | -1.32 |
|  |  | 15 | 0.42 | 4.25 | 10.29 | 14.87 | -1.45 | -10.60 | -4.22 | -7.95 |
|  |  | 20 | 0.49 | 3.44 | 13.25 | 18.32 | -1.75 | -11.59 | -5.90 | -10.26 |
|  |  | 25 | 0.56 | 3.22 | 16.10 | 22.24 | -1.87 | -13.58 | -7.71 | -15.89 |
|  |  | 30 | 0.75 | 5.23 | 19.33 | 29.41 | -2.23 | -17.22 | -8.92 | -16.56 |
|  |  | 35 | 0.94 | 5.31 | 22.03 | 35.27 | -2.59 | -17.88 | -9.82 | -18.87 |
|  |  | 40 | 1.05 | 4.88 | 22.98 | 50.30 | -3.49 | -29.47 | -9.58 | -25.17 |
|  | Targeted | 5 | 0.22 | 2.68 | 3.58 | 6.42 | -0.66 | -6.62 | -1.81 | 0.33 |
|  |  | 10 | 0.26 | 2.40 | 6.71 | 8.66 | -1.02 | -8.28 | -3.01 | -0.99 |
|  |  | 15 | 0.35 | 2.57 | 9.66 | 11.97 | -1.39 | -8.61 | -3.92 | -5.63 |
|  |  | 20 | 0.54 | 4.60 | 12.93 | 19.22 | -1.57 | -12.25 | -5.66 | -10.26 |
|  |  | 25 | 0.63 | 4.60 | 15.83 | 23.76 | -1.99 | -14.57 | -7.53 | -16.23 |
|  |  | 30 | 0.77 | 4.29 | 18.80 | 26.94 | -2.17 | -15.89 | -8.86 | -16.23 |
|  |  | 35 | 0.88 | 3.97 | 21.37 | 32.47 | -2.53 | -16.89 | -9.82 | -18.54 |
|  |  | 40 | 1.06 | 6.43 | 22.58 | 50.89 | -3.13 | -31.46 | -9.46 | -24.50 |
| Workforce | Random | 5 | 0.15 | 2.04 | 3.49 | 3.70 | -0.06 | -1.66 | -2.23 | -1.99 |
|  |  | 10 | 0.09 | 0.47 | 6.94 | 4.11 | -0.54 | -4.30 | -3.19 | -1.66 |
|  |  | 15 | 0.12 | -0.30 | 9.94 | 7.08 | -0.84 | -3.64 | -4.58 | -6.62 |
|  |  | 20 | 0.34 | 1.69 | 13.75 | 12.23 | -1.33 | -7.62 | -6.20 | -9.93 |
|  |  | 25 | 0.62 | 1.36 | 16.35 | 17.54 | -1.63 | -9.60 | -7.71 | -15.56 |
|  |  | 30 | 0.74 | 0.37 | 19.19 | 21.45 | -1.75 | -10.93 | -8.55 | -17.55 |
|  |  | 35 | 1.22 | 1.92 | 22.19 | 31.27 | -2.95 | -19.21 | -9.52 | -17.55 |
|  |  | 40 | 1.35 | 1.47 | 23.52 | 43.94 | -3.37 | -26.82 | -10.12 | -24.50 |
|  | Targeted | 5 | 0.09 | 1.38 | 3.66 | 2.75 | -0.12 | -1.32 | -2.35 | -1.66 |
|  |  | 10 | 0.09 | 0.83 | 6.78 | 5.06 | -0.54 | -8.28 | -3.92 | 2.98 |
|  |  | 15 | 0.24 | 0.18 | 9.73 | 8.32 | -1.08 | -9.27 | -4.46 | -2.32 |
|  |  | 20 | 0.29 | 1.38 | 13.61 | 13.01 | -1.75 | -10.93 | -6.08 | -4.30 |
|  |  | 25 | 0.27 | 0.48 | 16.25 | 18.13 | -1.99 | -16.23 | -7.29 | -8.61 |
|  |  | 30 | 0.38 | 0.54 | 19.19 | 21.47 | -2.71 | -16.23 | -8.61 | -11.26 |
|  |  | 35 | 0.61 | 2.14 | 21.73 | 33.89 | -3.01 | -21.85 | -9.64 | -18.87 |
|  |  | 40 | 0.74 | 1.94 | 23.31 | 45.14 | -3.80 | -33.77 | -9.34 | -18.21 |
| In-Home | Random | 5 | -0.08 | 0.73 | 0.21 | -1.22 | -0.24 | 0.00 | -1.08 | 0.99 |
|  |  | 10 | -0.20 | 1.05 | -0.57 | 3.28 | -0.36 | -3.97 | 1.93 | -0.33 |
|  |  | 15 | -0.06 | 2.34 | -0.24 | 1.36 | -0.78 | -4.97 | 0.18 | 1.99 |
|  |  | 20 | -0.17 | -0.33 | 0.00 | -0.01 | -0.18 | -1.32 | 3.43 | -1.66 |
|  |  | 25 | -0.51 | -0.82 | -0.42 | -4.34 | -0.66 | 1.66 | 1.33 | 0.66 |
|  |  | 30 | -0.52 | -1.27 | 0.11 | -7.46 | -0.12 | 1.99 | -0.72 | 5.63 |
|  |  | 35 | -0.76 | -1.72 | -0.61 | -3.24 | -0.24 | 1.66 | 1.39 | 0.33 |
|  |  | 40 | -0.49 | -1.17 | -0.04 | -6.53 | -0.48 | 0.99 | 0.30 | 0.00 |
